# Inhibition of the STAT3 Signaling Pathway Contributes to the Anti-Melanoma Activities of Shikonin

**DOI:** 10.3389/fphar.2020.00748

**Published:** 2020-05-27

**Authors:** Hui-Hui Cao, Dong-Yi Liu, Ye-Cai Lai, Yu-Yao Chen, Lin-Zhong Yu, Meng Shao, Jun-Shan Liu

**Affiliations:** ^1^Traditional Chinese Pharmacological, Third Level Research Laboratory of State Administration of Traditional Chinese Medicine, School of Traditional Chinese Medicine, Southern Medical University, Guangzhou, China; ^2^Guangdong Provincial Key Laboratory of Chinese Medicine Pharmaceutics, School of Traditional Chinese Medicine, Southern Medical University, Guangzhou, China; ^3^Guangzhou BaiYunShan Pharmaceutical General Factory, Guangzhou BaiYunShan Pharmaceutical Holdings Co., Ltd., Guangzhou, China

**Keywords:** shikonin, melanoma, apoptosis, migration, invasion, STAT3

## Abstract

**Background:**

Malignant melanoma is an extremely aggressive and metastatic cancer, and highly resistant to conventional therapies. Signal transducer and activator of transcription 3 (STAT3) signaling promotes melanoma development and progression, which has been validated as an effective target in melanoma treatment. Natural naphthoquinone shikonin is reported to exert anti-melanoma effects. However, the underlying mechanisms have not been fully elucidated.

**Purpose:**

This study aims to evaluate the anti-melanoma activities of shikonin and explore the involvement of STAT3 signaling in these effects.

**Methods:**

Zebrafish tumor model was established to evaluate the anti-human melanoma effects of shikonin *in vivo*. MTT assay and colony formation assay were employed to investigate the anti-proliferative effects of shikonin on human melanoma A375 and A2058 cells. Flow cytometry was used to analyze cell cycle distribution and apoptosis induction. Wound healing assay and Transwell chamber assay were conducted to examine the cell migratory and invasive abilities. Immunofluorescence assay was used to observe F-actin, Tubulin, and STAT3 localization. Western blotting was used to determine the expression levels of proteins associated with apoptosis and key proteins in the STAT3 signaling pathway. Immunoblotting was performed in DSS cross-linked cells to determine the homo-dimerization of STAT3. Gelatin zymography was employed to evaluate the enzymatic activity of MMP-2 and MMP-9. Transient transfection was used to overexpress STAT3 in cell models.

**Results:**

Shikonin suppressed melanoma growth in cultured cells and in zebrafish xenograft models. Shikonin induced melanoma cells apoptosis, inhibited cell migration and invasion. Mechanistic study indicated that shikonin inhibited the phosphorylation and homo-dimerization of STAT3, thus reduced its nuclear localization. Further study showed that shikonin decreased the levels of STAT3-targeted genes Mcl-1, Bcl-2, MMP-2, vimentin, and Twist, which are involved in melanoma survival, migration, and invasion. More importantly, overexpression of constitutively active STAT3 partially abolished the anti-proliferative, anti-migratory, and anti-invasive effects of shikonin.

**Conclusion:**

The anti-melanoma activity of shikonin is at least partially attributed to the inhibition on STAT3 signaling. These findings provide new insights into the anti-melanoma molecular mechanisms of shikonin, suggesting its potential in melanoma treatment.

## Introduction

Malignant melanoma is the most dangerous type of skin cancer. Although it represents less than 5% of the skin cancer cases, it accounts for 60% to 80% of all skin cancer-related deaths (Bandarchi et al., 2013; Orgaz and Sanz-Moreno, 2013). One of the main reasons for the lethality of melanoma is its rapid progression towards metastasis. The 5-year survival rate associated with the metastasis form of melanoma varies from 5% to 19% (Cummins et al., 2006; Bandarchi et al., 2013; Maverakis et al., 2015). Available therapeutic agents, such as dacarbazine (alkylating agents), vemurafenib/dabrafenib/encorafenib (BRAF V600E inhibitors), interferon alfa-2b/interleukin-2, ipilimumab (antibodies against CTLA-4), pembrolizumab/nivolumab (antibodies target PD-L1/PD-1), alimogene laherparepvec (oncolytic vaccines), have significantly improved patient survival (Zhu et al., 2016; Millet et al., 2017). However, adverse drug reactions, low response rates, and especially, drug resistances are major issues of the above agents in curing melanoma (Zhu et al., 2016; Millet et al., 2017). Therefore, other treatment strategies are still required.

Signal transducers and activators of transcription 3 (STAT3) is a member of STATs family that transmitting the signals from plasma membrane to the nucleus, where they ultimately lead to the regulation of target genes involved in cell differentiation, proliferation, survival, inflammatory response, angiogenesis, metastasis, and immune response (Carpenter and Lo, 2014). Inappropriately and persistently activated STAT3 have been estimated to occur in approximately 70% of hematopoietic and solid malignancies, including melanoma (Johnston and Grandis, 2011; Laudisi et al., 2018). The levels of activated STAT3 have been shown to correlate with a poor clinical prognosis in several of these cancers (Wu et al., 2016). As to melanoma, STAT3 activation plays a vital role in melanoma progression and metastasis (Niu et al., 2002a; Niu et al., 2002b). Abrogation of the STAT3 signaling cascade in melanoma caused growth inhibition, apoptosis, and impaired tumor growth and metastasis in animal models (Cao et al., 2014; Cao et al., 2016). Targeting STAT3 has been considered as a potential therapeutic strategy for melanoma treatment. Indeed, several STAT3 inhibitors showed promising results in early-phase clinical trials (Huynh et al., 2019). However, no STAT3 inhibitor has been approved for melanoma treatment.

Shikonin is a natural naphthoquinone isolated from traditional Chinese medicine *Zicao*, the dried root of *Arnebia euchroma* (Royle) Johnst. or *Arnebia guttata* Bunge. *Zicao* is traditionally used to treat a variety of inflammatory and infectious diseases, particularly in treating skin diseases, such as external wounds, burns, or dermatitis (Andujar et al., 2013). As the main efficacy component of *Zicao*, shikonin was reported to exert multiple biological functions, including anti-inflammatory, anti-bacterial, anti-viral, anti-oxidant, and anti-cancer activities (Andújar et al., 2013; Wang et al., 2019). In melanoma, shikonin was reported to inhibit human melanoma A375 cell growth, induce apoptosis, cell cycle arrest, and protective autophagy (Wu et al., 2004; Kretschmer et al., 2012; Liu et al., 2019b). Shikonin also inhibited murine melanoma B16 cell growth in an animal model (Zhao et al., 2018). However, the mechanisms responsible for its effect are not well studied. And although shikonin was reported to possess STAT3 inhibitory potency in human lung and breast cancer cells (Thakur et al., 2015; Guo et al., 2018; Tang et al., 2018a), its effect on STAT3 of melanoma have not been defined yet. In the current study, two human melanoma cell lines A375 and A2058 with constant STAT3 activation were used for studying the anti-melanoma activities of shikonin and the involvement of STAT3 in these effects.

## Materials and Methods

### Reagents

FITC Annexin V apoptosis detection kit was purchased from BD Biosciences (San Jose, CA, USA). FxCycle PI/RNase staining solution, BCA protein assay kit, Lipofectamine 2000 transfection reagent, and DSS were obtained from Thermo Fisher Scientific (Waltham, MA, USA). Antibodies against PARP, Caspase 3, STAT3, phospho-STAT3 (Tyr 705), Bcl-2, MMP-2, Twist, N-cadherin, vimentin, tubulin, and Alexa Fluor^®^ 488 Phalloidin were purchased from Cell Signaling Technology (Danvers, MA, USA). Goat anti-rabbit IgG, goat anti-mouse IgG, DyLight 594 goat anti-rabbit IgG, anti-Mcl-1, anti-Lamin B, and anti-β-actin were supplied by EarthOx Life Sciences (Millbrae, CA, USA). Cell tracker CM-DiI was supplied by Yeasen (Shanghai, China). Sorafenib was purchased from Aladdin (Shanghai, China). Other chemicals were obtained from Sigma-Aldrich (St. Louis, MO, USA). Shikonin was purchased from BeNa culture collection (Beijing, China, purity > 99%). The stock solution of 20 mM shikonin was prepared in dimethyl sulfoxide (DMSO).

### Cell Culture

Human melanoma A375 and normal human liver-derived cells (MIHA) were purchased from American Type Culture Collection (ATCC, USA), and human melanoma A2058 cells were obtained from Cell Lines Service GmbH (CLS, Germany). All cell lines were maintained at 37°C and 5% CO_2_ in a humidified atmosphere in high glucose Dulbecco's modified Eagle's medium (DMEM, GIBCO, USA), supplemented with 10% fetal bovine serum (FBS, GIBCO, USA) and 1% penicillin/streptomycin (GIBCO, USA).

### Xenotransplantation in Zebrafish Embryos

Transgenic zebrafish Tg (Fli1:GFP) were kindly provided by the key laboratory of Zebrafish Modeling and Drug Screening for Human Diseases Institute at Southern Medical University (Guangzhou, China). Establishment of zebrafish xenograft tumor model was performed as previously described (Liu et al., 2019a). Briefly, CM-DiI (2 μM) labeled A375 and A2058 cells were micro-injected into the yolk sac of 2 days post fertilization (dpf) embryos. Then these embryos were treated with indicated concentration of shikonin (0.015, 0.0625, 0.25 μM) or sorafenib (0.5 μM) for 48 h, and observed by a fluorescence microscopy at 0, 24, and 48 h treatment (Olympus MVX10, Olympus, Japan).

### Hematoxylin and Eosin Staining

Paraformaldehyde-fixed zebrafish embryos were embedded with paraffin and cut into 5-μm-thin sections. Then they were deparaffinized, stained with hematoxylin and eosin (H&E). Tumor cells in the yolk sac were observed using a light microscope.

### Cell Viability Assay and Colony Formation Assay

The cytotoxicity of shikonin in A375 and A2058 cells was detected by MTT assay as described previously (Cao et al., 2014).

Colony formation assay was also employed to determine the anti-proliferative effects of shikonin on melanoma. Cells seeded in six-well plates (5000/well) were treated with indicated concentrations of shikonin for 24 h. Then, cells were washed with PBS and cultured in fresh medium for 10 days to allow colony formation. The resulting colonies were formalin-fixed and stained with crystal violet. Finally, crystal violet was removed, colonies were washed, and images were photographed.

### Detection of Cell Cycle Distribution and Apoptosis

A375 and A2058 cells (3 × 10^5^/well) were seeded in six-well plates overnight and treated with shikonin at different concentrations for 24 h. Then, cells were harvested, washed with cold PBS, and fixed with 70% ethanol at 4°C overnight. After that, cells were stained with FxCycle PI/RNase staining solution. Cellular DNA was analyzed by flow cytometry (CytoFLEX, Beckman Coulter, CA, USA) using ModFit LT 3.1 software (Verity Software House, USA).

Shikonin-induced cell apoptosis was measured by a FITC Annexin V apoptosis detection kit according to the manufacturer's protocol (Cao et al., 2014).

### *In Vitro* Cell Migration and Invasion Assay

The effect of shikonin on cell migration was evaluated by wound healing assay and Transwell migration assay as described previously (Cao et al., 2014; Cao et al., 2015; Cao et al., 2016).

Transwell invasion assay was tested using a BD BioCoat™ Matrigel™ invasion chamber (8 μm pore size, Corning, NY, USA) according to the manufacturer's protocol.

### Immunofluorescence Assay

Cells grown in a glass bottom dish were treated with shikonin for 24 h. Then, cells were fixed with 4% paraformaldehyde, permeabilized with 0.2% Triton X-100, and blocked with 5% BSA in PBS. After that, cells were incubated overnight at 4°C with specific primary antibodies against Tubulin. Subsequently, cells were incubated with secondary antibody labeled with DyLight 594 for 1 h at room temperature in darkness, and then stained with Alexa Fluor^®^ 488 Phalloidin 15 min at room temperature. Images of the cell signal were observed under a confocal microscope (LSM800, Carl Zeiss, Oberkochen, Germany).

To observe the localization of STAT3, cells were incubated with primary STAT3 antibody overnight at 4°C, followed by incubation of secondary antibody labeled with DyLight 594 for 1 h at room temperature in darkness. After counterstained with DAPI, images of the cell signal were observed under a confocal microscope (LSM800, Carl Zeiss, Oberkochen, Germany).

### Western Blot Analysis

Subcellular fractionation, whole cell lysate preparation and Western blot analysis were performed as described previously (Cao et al., 2014).

### Gelatin Zymography

The enzymatic activities of MMP-2 and MMP-9 were determined by gelatin zymography as described previously (Cao et al., 2014; Cao et al., 2016).

### Detection of STAT3 Dimer

Cells treated with shikonin were collected and suspended in PBS. The crosslinker disuccinimidyl suberate (DSS, 0.5 mM) was added to cells and reacted for 30 min at room temperature. Subsequently, 20 mM Tris-HCl (pH 7.4) was added and incubation for 15 min at room temperature to quench the reactions. Finally, cell lysates were separated by 6% SDS-PAGE and immunoblotted with an anti-STAT3 antibody.

### Plasmid Transient Transfection

Constitutively active STAT3 expression construct STAT3-C Flag pRc/CMV was obtained from Addgene (USA). Transfection of STAT3C plasmids into melanoma cells was conducted by lipofectamine 2000 following manufacturer's protocol. Empty pcDNA3.0 plasmid was used as mock transfectant. Cells were transfected with plasmids for 24 h before functional assays were carried out.

### Statistical Analysis

Statistical analysis was performed by the Graphpad Prism 5.0 software (Graphpad software Inc., CA, USA). All data were presented as means ± S.D. from at least three independent experiments. *P* < 0.05 was considered as statistically significant.

## Results

### Shikonin Inhibited the Growth of Melanoma Cells in Zebrafish Tumor Model

**Figure 1:**
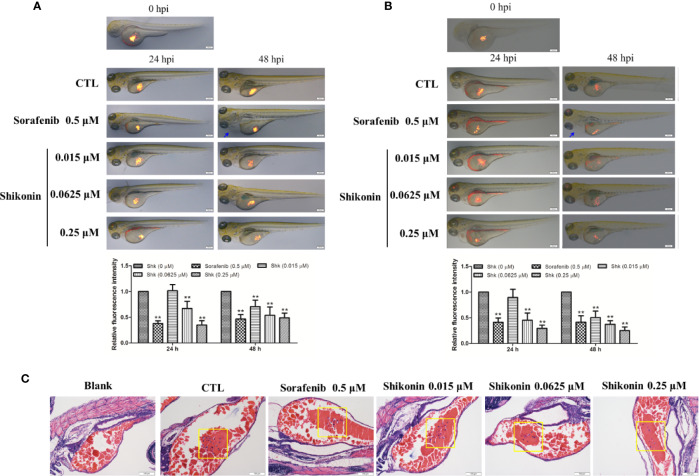
Shikonin inhibited melanoma tumor growth in zebrafish xenograft model. Fluorescently labeled **(A)** A375 or **(B)** A2058 cells were microinjected into the yolk sac of 2 dpf zebrafish embryos. These zebrafish were then transferred randomly to 24-well plates, 10 embryos per well with 0.5 ml of embryo medium containing indicated concentrations of shikonin or sorafenib for a treatment period of 48 h. Representative images of embryos were shown as merged bright-field and fluorescent images at 0, 24, and 48 h. Relative fluorescence intensity were analyzed by Image J software. Data were shown as mean ± SD from three independent experiments, **P < 0.01. Blue arrows indicated pericardia. **(C)** H&E staining of A375 cell transplanted zebrafish. Tumor cells in yolk were shown (yellow squares).

Zebrafish tumor model is an ideal tool in melanoma medicine discovery (Lally et al., 2007; Van Rooijen et al., 2017). To evaluate the anti-melanoma activity of shikonin, a zebrafish melanoma model was established by microinjection of CM-DiI-stained melanoma cells, and then, these embryos were treated with indicated concentrations of shikonin or sorafenib. The inhibitory effects of shikonin were evaluated by the observation of red fluorescence. As shown in **Figure 1**, consistence with sorafenib treatment, red fluorescence in zebrafish yolk was dose-dependently reduced after shikonin treatment. The same result was obtained in A2058 cells implanted embryos. These results indicate that shikonin inhibits the tumor formation *in vivo*.

### Shikonin Reduced Viability and Induced Apoptosis in Melanoma Cells

**Figure 2:**
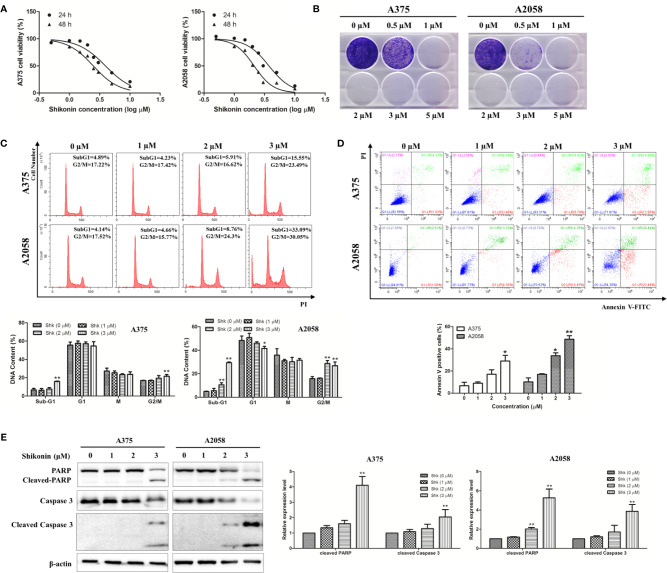
Shikonin reduced viability and induced apoptosis in human melanoma cells. A375 and A2058 cells were treated with indicated concentrations of shikonin. **(A)** Cell viability was measured by MTT assay after 24 or 48 h treatment. **(B)** Represented photos showed the effect of shikonin on clonogenic survival of A375 and A2058 cells. **(C)** Cell cycle phases of the treated cells were evaluated by flow cytometry and analyzed by ModFit software. The DNA content of cells at different cell phase was presented as the mean ± SD of three independent experiments. *P < 0.05 and **P < 0.01. **(D)** Apoptosis was analyzed by flow cytometry after 24 h treatment. The percentage of apoptotic cells was presented as the mean ± SD of three independent experiments. *P < 0.05 and **P < 0.01. **(E)** Cleavages of PARP and caspase-3 were detected by Western blot analysis after 24 h treatment (left panel) and relative expression levels (right panel) were analyzed by Image J software. Data were shown as mean ± SD from three independent experiments, *P < 0.05, **P < 0.01.

We then investigated the inhibitory effects of shikonin in A375 and A2058 cells *in vitro*. As shown in **Figure 2A**, shikonin treatment (0.5, 1, 1.5, 2, 2.5, 3, 5, 10 μM) decreased the viabilities of A375 and A2058 cells in time- and dose-dependent manners, with IC_50_ values of 3.938 ± 0.258 μM and 2.481 ± 0.1 μM in A375 cells, and 3.735 ± 0.236 μM and 2.124 ± 0.085 μM in A2058 cells after 24 and 48 h treatments, respectively. We also found that the cytotoxicity of shikonin on normal human liver-derived cells (MIHA) was much lower than that on human melanoma cells (see **Supplementary Figure 1**). Data from colony formation assay further confirmed that shikonin dose-dependently decreased the proliferation of A375 and A2058 cells (**Figure 2B**).

To determine whether shikonin-induced cell growth inhibition was associated with cell cycle arrest, flow cytometry was performed to analyze the cell cycle. As shown in **Figure 2C**, the percentage of cells in sub-G1 phase was increased dose-dependently by shikonin treatment, and increased cells in G2/M phase was also observed.

Since increase in sub-G1 phase of the cell cycle may be caused by apoptosis, we then examined the capacity of shikonin treatment to induce apoptosis in A375 and A2058 cells. Data from annexin V/PI double staining assay showed that shikonin dose-dependently increased the percentage of apoptotic cells from 6.64 ± 5.43% to 29.71 ± 8.80% in A375 cells, and from 10.17 ± 6.24% to 45.98 ± 5.53% in A2058 cells after 24 h treatment (**Figure 2D**).

To confirm apoptosis induction, we examined the presence of PARP/caspase-3 cleavage in shikonin-treated melanoma cells. As shown in **Figure 2E**, cleaved PARP was evident in both A375 and A2058 cells, and the expression levels of cleaved-caspase-3 was also increased after treated with shikonin for 24 h. These results indicate that shikonon inhibits cell growth and induces apoptosis in melanoma cells.

### Shikonin Impaired Melanoma Cell Metastasis *In Vitro*

**Figure 3:**
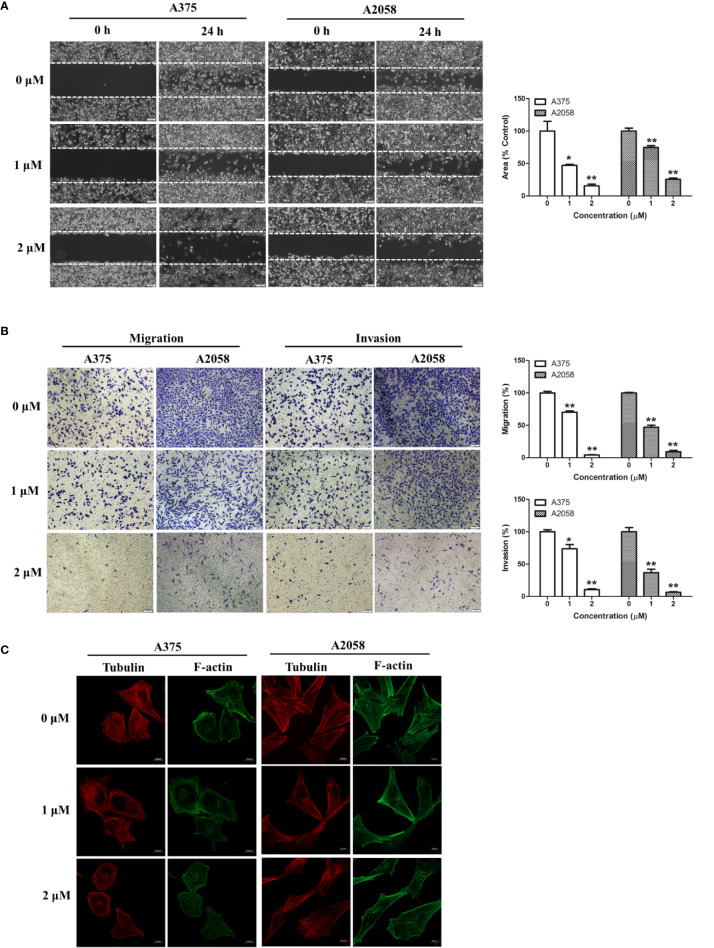
Shikonin inhibited melanoma cell migration and invasion. **(A)** A single scratch was created in the confluent monolayer of A375 or A2058 cells. The scratch was photographed at 0 and 24 h after shikonin treatment (left) and relative migrated areas (right) were analyzed by Image J software. **(B)** Cells were allowed to migrate through membranes without or with Matrigel basement in the presence of indicated concentration of shikonin. Representative photographs of migrated/invasive cells (left) and quantification of these cells (right) were shown. **(C)** Cells were treated with shikonin for 24 h and changes in Tubulin and F-actin were visualized using confocal microscope. Data were shown as mean ± SD from three independent experiments, *P < 0.05 and **P < 0.01.

We used wound healing assay and migration chamber assay to investigate the effects of shikonin on melanoma cell migration. After 24 h incubation, cells were found to migrate to the wound area, but shikonin at 1 μM strongly inhibited A375 and A2058 cell migration (**Figure 3A**). In the migration chamber assay, the number of cells transferred to the lower membrane of the chamber in shikonin-treated groups was fewer than that in vehicle control groups (**Figure 3B**), which further confirmed the anti-migratory ability of shikonin in melanoma. Shikonin also exerted anti-invasive activity in melanoma cells in a Transwell invasion model (**Figure 3B**).

Cellular movement is mainly orchestrated by the actin and microtubule cytoskeleton, which provide cell shape and maintain cellular structure and polarization (Akhshi et al., 2014). We then observed the structure of tubulin and actin cytoskeleton in shikonin-treated melanoma cells by fluorescence microscopy. As shown in **Figure 3C**, untreated melanoma cells displayed broad F-actin extensions along the membrane and an intact microtubule network. Shikonin treatment resulted in pronounced changes in the cytoskeletal architecture in melanoma cells. F-actin stress fiber patterns were remarkably reduced, and located within the cytoplasm instead of the membrane of the cells, while microtubule network was disrupted and reduced. Shikonin in these experiments did not inhibit cell proliferation (**Figure 2A**).

### Shikonin Inhibits STAT3 Signaling

**Figure 4:**
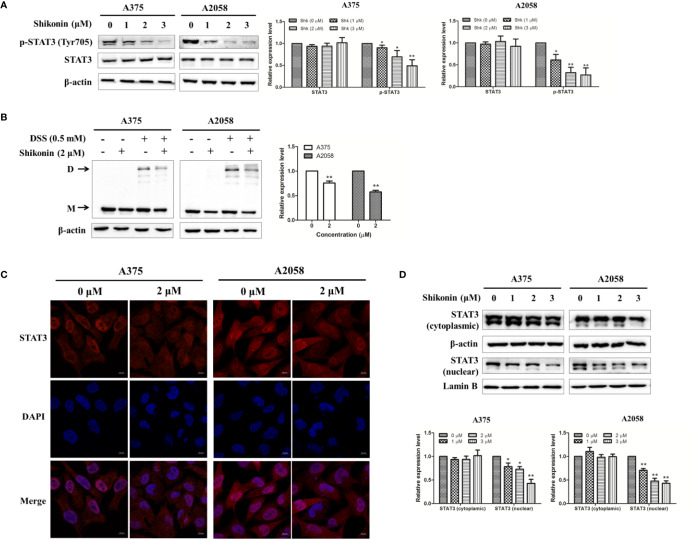
Shikonin inhibited STAT3 signaling pathway in melanoma cells. **(A)** Cells were treated with indicated concentration of shikonin for 24 h, expression levels of STAT3 and p-STAT3 were determined by the Western blot analysis (upper), and relative expression levels were analyzed by Image J software (bottom). **(B)** Cells were treated with 2 μM shikonin for 24 h, and then incubated with DSS. Changes in STAT3 dimerization were evaluated by immunoblotting. The arrows indicated the dimer **(D)** and the monomer (M) STAT3. The representative results (upper) and the relative expression levels of dimer STAT3 (bottom) were shown. **(C, D)** Cells were treated with indicated concentration of shikonin for 24 h, and then the intracellular distribution of STAT3 was analyzed by immunofluorescence assay **(C)** and immunoblotting **(D)**. Relative expression levels of cytosolic and nuclear STAT3 were also shown (bottom). Data were shown as mean ± SD from three independent experiments, *P < 0.05 and **P < 0.01.

It has been reported that constitutive activation of the STAT3 signaling pathway promotes melanoma cell growth, survival, migration, invasion, and allows cancer metastasis (Niu et al., 2002a; Niu et al., 2002b). Therefore, we determined the effects of shikonin on the activation/phosphorylation of STAT3 in human melanoma cells. As shown in **Figure 4A**, the phosphorylation of STAT3 at the tyrosine 705 (Tyr705) site was dose-dependently inhibited by shikonin in A375 and A2058 cells, while shikonin did not affect the total STAT3 protein levels at the same concentration.

Tyrosine phosphorylation of STAT3 at Tyr705 initiates STAT3 homodimerization and nuclear translocation. So we examined whether shikonin could affect the dimerization and nuclear translocation of STAT3. As shown in **Figure 4B**, incubation with a cross linker DSS increased STAT3 dimerization, while pretreatment with shikonin reduced the expression level of STAT3 dimer. The cellular-distribution of STAT3 was determined by immunofluorescence assay and immunoblotting. Data showed that shikonin treatment significantly reduced nuclear STAT3 levels in melanoma cells (**Figures 4C, D**).

### STAT3 Partially Mediates the Effects of Shikonin on Cell Viability, Migration, and Invasion

**Figure 5:**
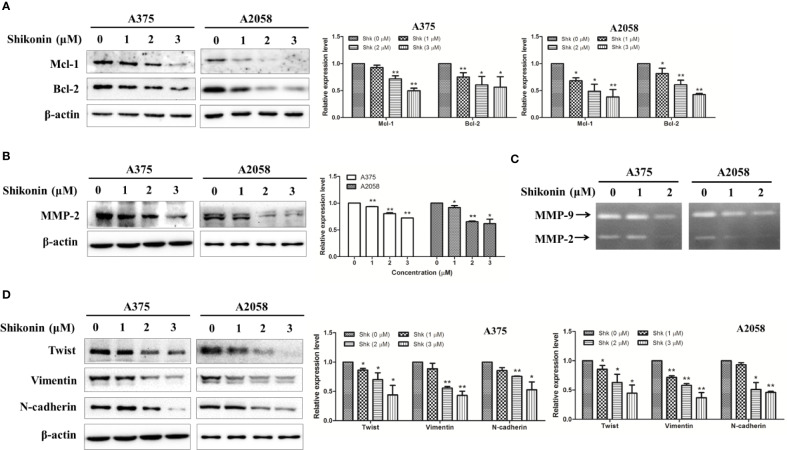
Shikonin down-regulated protein levels and inhibited enzymatic activities of STAT3-targeted molecules in melanoma cells. A375 and A2058 cells were treated with indicated concentrations of shikonin for 24 h, and then total cell lysates were collected, expression levels of **(A)** Mcl-1, Bcl-2, **(B)** MMP-2, **(D)** Twist, Vimentin, and N-cadherin were evaluated by Western blot analysis (left) and relative expression levels were analyzed by Image J software (right). Data were shown as mean ± SD from three independent experiments, *P < 0.05 and **P < 0.01. **(C)** The enzymatic activities of MMP-2 and MMP-9 were determined by gelatinase zymography.

We next determined the effects of shikonin on STAT3-targeted molecules in human melanoma cells. Mcl-1 and Bcl-2 are the target genes of STAT3 involving in cell survival, and MMP-2 and MMP-9 are involved in cell migration and invasion (Carpenter and Lo, 2014). As shown in **Figure 5**, shikonin dose-dependently decreased the protein levels of Mcl-1, Bcl-2, and MMP-2. The gelatinase activities of MMP-2 and MMP-9 were also inhibited by shikonin, in both A375 and A2058 cells. Twist is a transcription factor that directly regulated by STAT3, and is reported to be able induce epithelial-to-mesenchymal transition (EMT), a biochemical changes in epithelial cells that usually includes enhanced migratory and invasive capacities (Lo et al., 2007). Vimentin and N-cadherin are key regulators in EMT process (Lamouille et al., 2014). Western blot analysis showed that shikonin also caused the downregulation of Twist, Vimentin, and N-cadherin.

**Figure 6:**
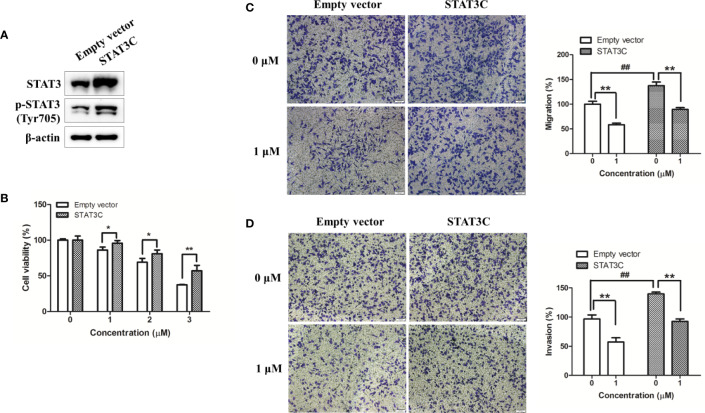
Overexpression of STAT3 in human melanoma A375 cells reduced shikonin-mediated cell growth, migration, and invasion inhibition. A375 cells were transiently transfected with an empty vector or a STAT3C-expressing construct for 24 h, and then **(A)** the expression level of STAT3 in A375-STAT3C cells and A375-EV cells were examined by immunoblotting. **(B–D)** A375-EV or A375-STAT3C cells were incubated with shikonin for 48 h or 24 h, and then **(B)** cell proliferation, **(C)** cell migratory, and **(D)** cell invasive abilities were measured. Representative photographs of migrated cells (left) and quantification of these cells (right) were shown. Data were mean ± SD from three independent experiments, ^##^*P* < 0.01, *P < 0.05, and **P < 0.01.

To further determine the involvement of STAT3 in shikonin-mediated anti-melanoma action, we overexpressed STAT3 in A375 cells by transiently transfected with a constitutively active STAT3 plasmid or an empty vector (EV). After 24 h transfection, the expression levels of STAT3 and p-STAT3 (Try705) were increased remarkably (**Figure 6A**). As expected, the viability inhibition rate by shikonin was significantly decreased in STAT3C-expressing A375 cells (A375-STAT3C) when compared with that on empty vector-transfected A375 cells (A375-EV) (**Figure 6B**). STAT3C overexpression in A375 cells also remarkably attenuated 1 μM of shikonin-induced cell migration and invasion inhibition (**Figures 6C, D**).

## Discussion

The use of natural compounds and medicinal based plants for health promotion and adjuvant therapy is becoming increasingly popular worldwide (Li et al., 2019; Liu et al., 2019c). *Zicao* has been used as an effective treatment for a variety of inflammatory and infectious diseases for a long history. Shikonin and its derivatives are identified as the main active components (Andújar et al., 2013; Wang et al., 2019). In this study, we examined the anti-melanoma activity of shikonin in cultured cells and transplanted zebrafish model. In agreement with the previous studies (Wu et al., 2004; Kretschmer et al., 2012; Liu et al., 2019b), shikonin inhibited melanoma cell proliferation, induced cell apoptosis, and cell cycle arrest *in vitro*. Furthermore, we found that shikonin exerted *in vivo* anti-melanoma activity in transplanted zebrafish model.

Zebrafish have emerged as a valuable animal model for a diverse spectrum of human diseases over the last decade. Compared with traditional vertebrate models, zebrafish represent unique advantages in relatively rapid life cycle, real-time imaging, and genetic manipulation. As to melanoma, zebrafish is a powerful model in studying melanocyte biology, identifying novel disease genes, and discovering new therapeutics that regulate melanocyte and melanoma development (Lally et al., 2007; Ceol et al., 2008; Van Rooijen et al., 2017). In this study, microinjection of melanoma cells to the yolk sack of 2 dpf embryos, a well-established tumor cell xenograft zebrafish model (Van Der Ent et al., 2014; Saltari et al., 2016; Pontes et al., 2017), was used to determine the *in vivo* anti-melanoma activity of shikonin. Our data showed that shikonin dose-dependently inhibited melanoma cell growth. Sorafenib, an oral multikinase inhibitor that approved by FDA for the treatment of several types of cancer, and also safe and feasible in melanoma patients (Monk et al., 2014; Wilson et al., 2016), was used as positive drug in this experiment. Sorafenib showed significant inhibitory effects in A375 and A2058 bearing zebrafish embryos (**Figure 1**). However, the toxicity of sorafenib was also observed. At 48 hpi, pericardial effusion was found in sorafenib-treated zebrafish (**Figure 1**, blue arrows). While shikonin exerted comparable anti-melanoma activity with sorafenib in zebrafish model and in cultured cells (**Figures 1** and **2A** and **Supplementary Figure 2**) but did not show any toxicity in zebrafish. These data indicate that shikonin is a promising anti-melanoma agent with relative low toxicity. And, injection cancer cells into the yolk sac is an ideal model for evaluation the anti-cancer efficacies and toxicities of large number of small molecules.

Shikonin has previously been shown to possess anti-metastatic effect in breast cancer and lung cancer (Jang et al., 2014a; Jang et al., 2014b; Thakur et al., 2015), but there is little information concerning its effects on melanoma metastasis. Here, we demonstrated that shikonin effectively inhibited melanoma cell migration and invasion. Shikonin also disrupted the shape and distribution of F-actin and α-tubulin, both of which are crucial modulators of the structural organization of cells and cell migration. Further study showed that shikonin downregulated the expression levels of EMT factors vimentin and N-cadherin. EMT is widely recognized as an important mechanism for cancer progression. These data indicate that multiple mechanisms are involved in shikonin-mediated melanoma cell migration and invasion inhibition. In the future, we will evaluate if shikonin inhibits melanoma metastasis and EMT in zebrafish model.

In the mechanistic study, we demonstrated that shikonin is a potent inhibitor of STAT3. More importantly, ectopic expression of STAT3 partially reversed the growth and migratory inhibition that caused by shikonin. These findings strongly suggest that inhibition of STAT3 signaling by shikonin may serve as an effective approach for melanoma treatment. It is of interest to note that several shikonin derivatives that target STAT3 have been identified, and some of them were proved to exert anti-cancer activities (Qiu et al., 2017a; Qiu et al., 2017b). After phosphorylation on tyrosine site 705, STAT3 homo-dimerized and translocate into the nucleus, where it mediates the transcription of target genes. Therefore, inhibition of STAT3 phosphorylation may result in decreased STAT3 homodimers and nuclear localization. In agreement with this, our results showed that the expression levels of dimer STAT3 and nuclear STAT3 were substantially reduced by shikonin treatment.

STAT3 is well recognized for favoring melanoma development and progression by regulating several genes. Our data showed that shikonin inhibited the expression levels of antiapoptotic proteins Bcl-2 and Mcl-1, and enzymatic activities of collagenases MMP-2 and MMP-9, which play vital roles in melanoma metastasis. Twist is a transcription factor that directly regulated by STAT3, and STAT3-mediated TWIST gene expression is responsible for cancer cell EMT (Lo et al., 2007). However, whether shikonin inhibits melanoma EMT and metastasis through STAT3/Twist inhibition needs to be further investigated. These results demonstrate that the suppressive capabilities of shikonin on melanoma cell survival, migration, and invasion may be attributed to the inhibition of STAT3-targeted Mcl-1, Bcl-2, MMP-2, MMP-9, vimentin, and Twist. It is reported that many other mechanisms like induced ROS generation, targeted p53 pathway, and ER stress were involved in the anti-melanoma action of shikonin (Wu et al., 2004; Liu et al., 2019b), we could not exclude the possibilities that shikonin could modulate other mechanisms that regulate cell response.

Constitutive STAT3 activation is usually mediated by upstream receptor tyrosine kinases JAKs or non-receptor tyrosine kinases, including Src and ABL (Huynh et al., 2019). So we tested the effect of shikonin on Src and JAK2. Data showed that shikonin inhibited the activation of JAK2, but did not apparently influence the expression levels of p-Src (see **Supplementary Figure 3**). These results suggest that inhibition of STAT3 may partially attribute to the inactivation of JAK2. Since the activation of tyrosine kinases is usually initiated by a diverse array of cytokines, growth factors, and peptide hormones binding to the receptors on cell surface (Huynh et al., 2019). We also found that shikonin can inhibit EGF-induced STAT3 phosphorylation in A375 cells (data not shown). And the effect of shikonin on EGFR/STAT3 is being explored.

Apart from STAT3, MAPK, and AKT signaling cascades are also constantly activated and promote melanoma progression (Yajima et al., 2012). So we evaluated the expression levels of p-p38, p-Erk, and p-Akt in shikonin-treated melanoma cells. Our data revealed that shikonin inhibited AKT phosphorylation, but dose- and time-dependently activated MAPK (see **Supplementary Figure 4**), which may hamper its anti-melanoma activity. The activation of Erk and p38 by shikonin was also observed in other cell models (Zhou et al., 2017; Huang et al., 2018). Many researches have studied the combination of shikonin with other therapies to treat cancer, which have enhanced the anti-cancer effects (Zhao et al., 2015; Matias et al., 2017; Ni et al., 2018; Tang et al., 2018b). Combined targeting of the MAPK pathway along with inhibitors of STAT3 was suggested to counteract STAT3-mediated resistance phenotypes in human melanoma cells (Vultur et al., 2014).Thus, shikonin may be a valuable candidate along with the specific inhibitor(s) of MAPK pathways to enhance the efficiency of melanoma treatments. However, specific inhibitors of Erk and p38 should be used to confirm the role of individual MAPKs on shikonin-mediated anti-melanoma activities.

Shikonin is high lipophilicity, which made it insolubility in water and affected its bioavailability. Hence, several formulation techniques for shikonin have been investigated, including liposome (Xia et al., 2013), composite fibers (Han et al., 2009), and microencapsulation (Huang et al., 2007). The solubility and bioavailability of shikonin have been significantly enhanced, and the toxicity was decreased by these strategies. Besides, shikonin presents favorable pharmacokinetic and pharmacodynamic profiles *in vivo* (Andújar et al., 2013). Prolonged exposure of shikonin to cancer cells was reported does not cause chemo-resistance (Wu et al., 2013). Collectively, shikonin could be developed as a potent anti-cancer agent.

In conclusion, our study shows that shikonin inhibits melanoma cell growth *in vitro* and in zebrafish melanoma model, induces cell apoptosis, suppresses cell migration and invasion. These effects are, at least in part, due to the inhibition of STAT3 signaling. Our findings provide novel insights into the anti-melanoma molecular mechanisms of shikonin, and further suggest a potential role of shikonin in melanoma treatment.

## Data Availability Statement

All datasets generated for this study are included in the article/**Supplementary Material**.

## Ethics Statement

The animal study was reviewed and approved by the Institutional Animal Care and Use Committee of Southern Medical University.

## Author Contributions

H-HC designed the study, carried out the majority the experiments and drafted the manuscript. D-YL performed several experiments and analyzed data. Y-YC participated in the zebrafish model experiment. Y-CL contributed to the reagents. J-SL, L-ZY, and MS conceived the study and participated in its design. All authors read and approved the final manuscript.

## Funding

This work was supported by the National Natural Science Foundation of China (81602997, 81402801), the China Postdoctoral Science Foundation (2016M590797), the Natural Science Foundation of Guangdong Province (2017A030313772, 2017A030306006, 2020A1515011239, 2020A1515010603), the Administration of Traditional Chinese Medicine of Guangdong Province (20181167), the Guangdong Province Universities and Colleges Pearl River Scholar Funded Scheme (GDHVPS2018), and the Guangzhou Science and Technology Project (201904010405, 201710010026).

## Conflict of Interest

Author Y-CL was employed by Guangzhou BaiYunShan Pharmaceutical Holdings Co., Ltd.

The remaining authors declare that the research was conducted in the absence of any commercial or financial relationships that could be construed as a potential conflict of interest.

## Abbreviations

STAT3, signal transducer and activator of transcription 3; dpf, days post fertilization; H&E, hematoxylin and eosin; DSS, disuccinimidyl suberate; EMT, epithelial-to-mesenchymal transition.
