# Supplementary material for: Inhibition of the STAT3 Signaling Pathway Contributes to the Anti-Melanoma Activities of Shikonin
Source: Front Pharmacol. 2020 May 27;11:748. doi: 10.3389/fphar.2020.00748 (PMC7267064; doi:10.3389/fphar.2020.00748)
Supplement: Supplementary file 1 [file DataSheet_1.pdf]

Inhibition of the STAT3 signaling pathway contributes to the anti-melanoma activities of shikonin

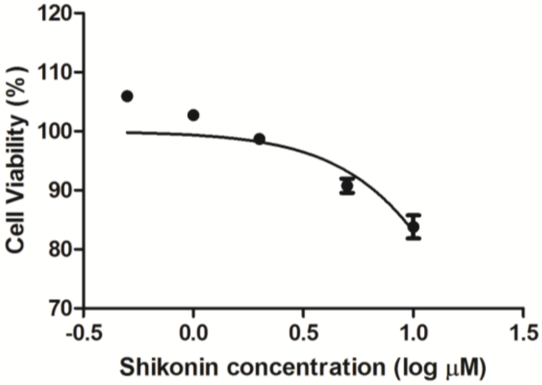

Figure S1 Cytotoxicity of shikonin on MIHA cells. Cells were treated with shikonin at 0.5, 1, 2, 5 and 10 μM for 24 h, and cell viability was determined by MTT assay and analyzed by Gaphpad prism software.

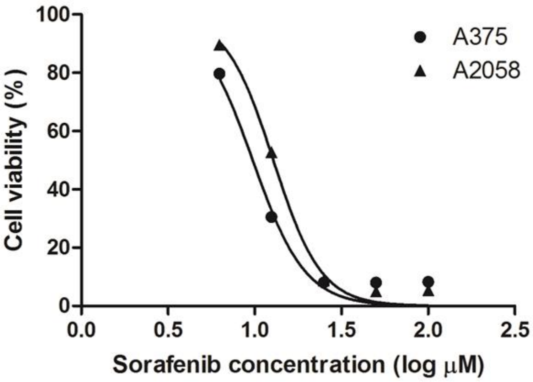

Figure S2 Cytotoxicity of sorafenib on melanoma cells. A375 and A2058 cells were treated with sorafenib at 6.25, 12.5, 25, 50, and 100 μM for 24 h, cell viability was determined by MTT assay and analyzed by Gaphpad prism software.

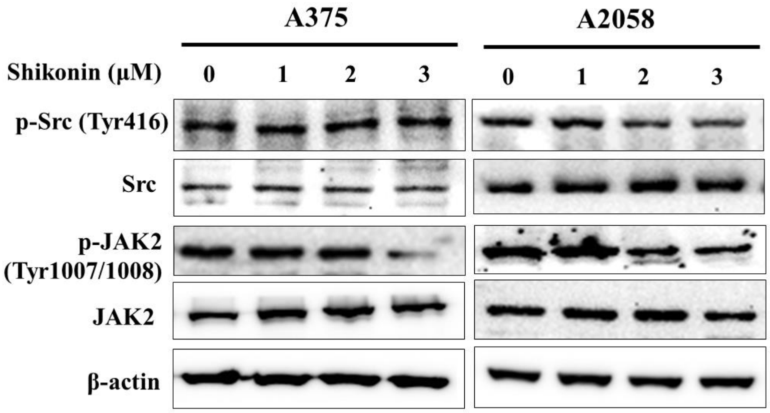

Figure S3 Effects of shikonin on Src and JAK2 phosphorylation. Melanoma cells were treated with shikonin for 24 h, immunoblotting was applied to evaluate the expression levels of p-Src and p-JAK2.

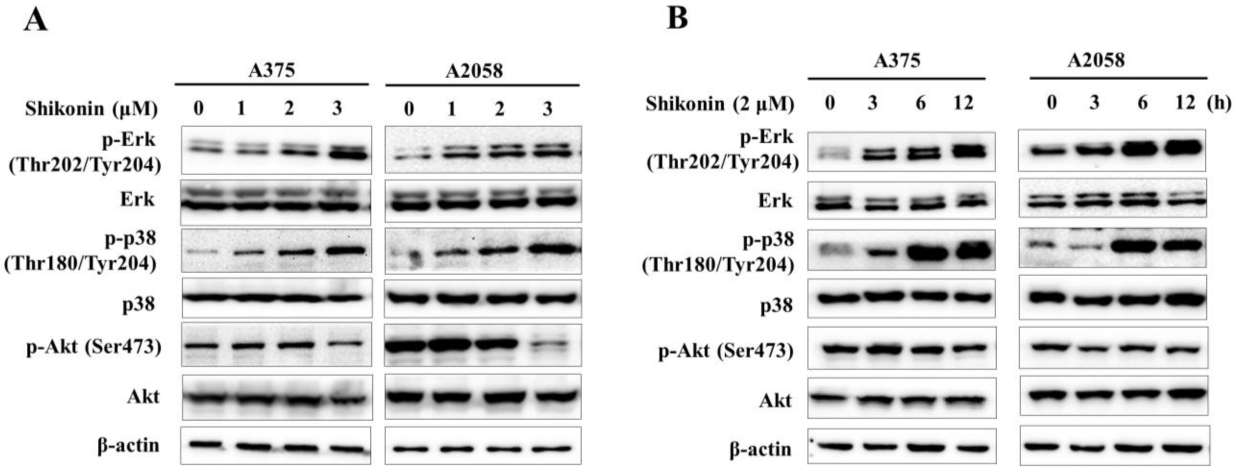

Figure S4 Effects of shikonin on the MAPK and AKT signaling pathway. A375 and A2058 cells were treated with various concentration of shikonin for 24 h or a fixed concentration (2 μM) for various durations, and then total cell lysates were extracted for Western blot analysis by using specific antibodies.
