# Supplementary figures and images for: Inhibition of the STAT3 Signaling Pathway Contributes to the Anti-Melanoma Activities of Shikonin
Source: Front Pharmacol. 2020 May 27;11:748. doi: 10.3389/fphar.2020.00748 (PMC7267064; doi:10.3389/fphar.2020.00748)

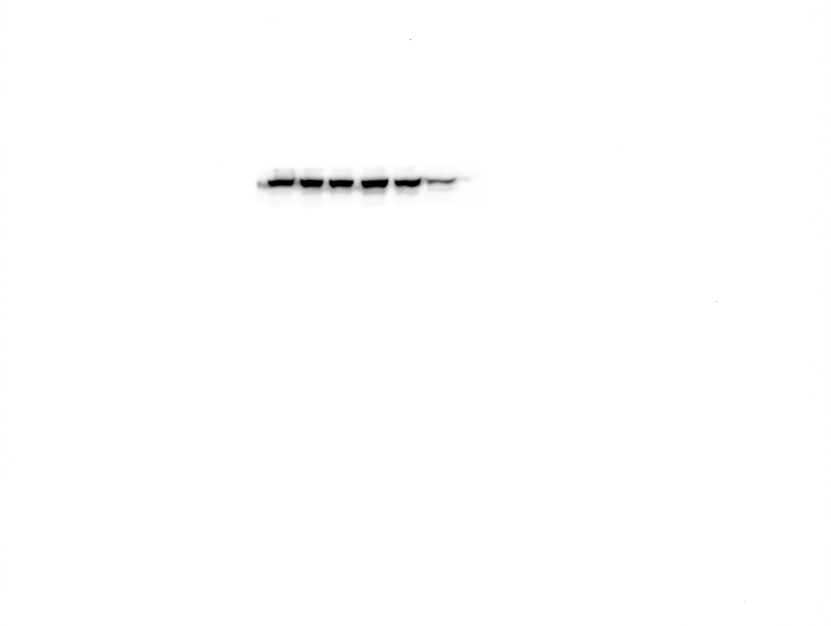

Supplement: Supplementary file 2 [file DataSheet_2.zip › Uncropped images/A2058 cells/Akt (different dosages).tif]

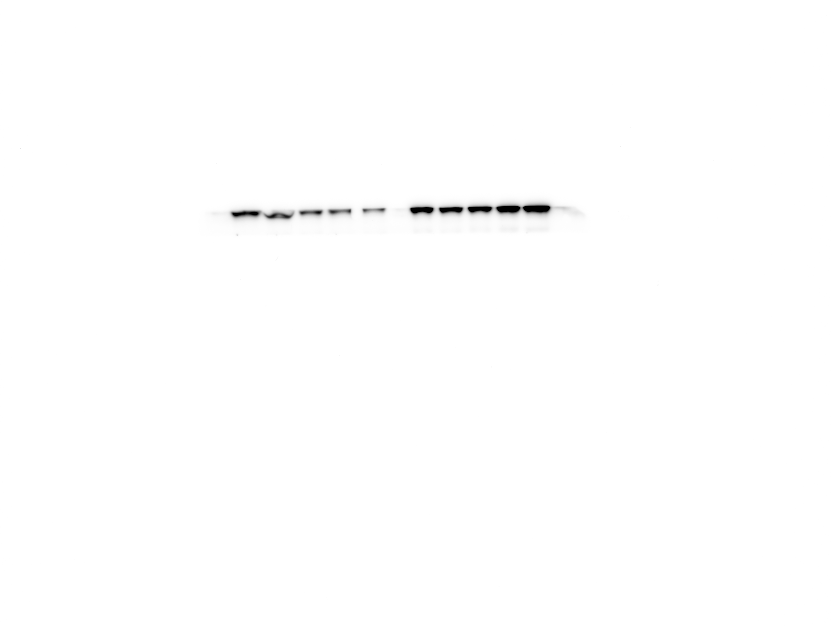

Supplement: Supplementary file 2 [file DataSheet_2.zip › Uncropped images/A2058 cells/Akt (different treatment durations).tif]

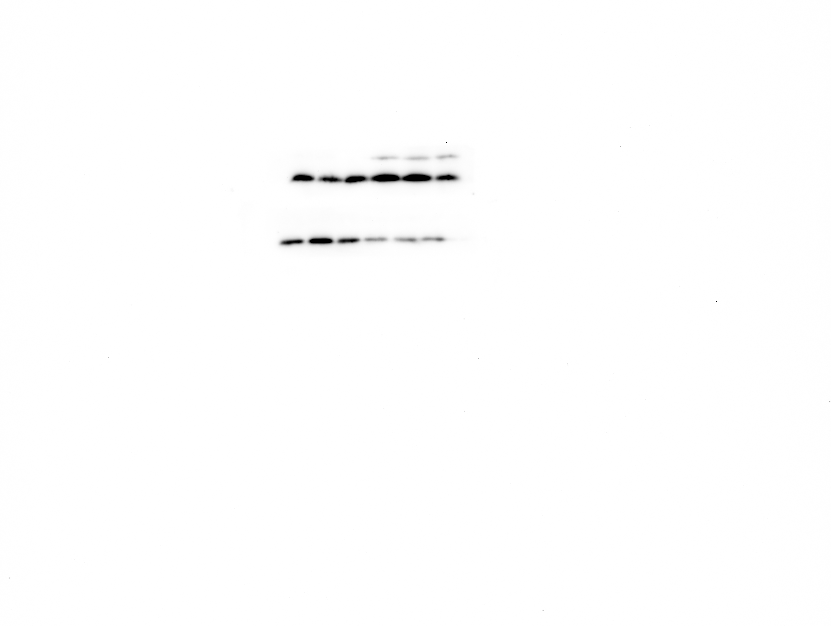

Supplement: Supplementary file 2 [file DataSheet_2.zip › Uncropped images/A2058 cells/Bcl-2.tif]

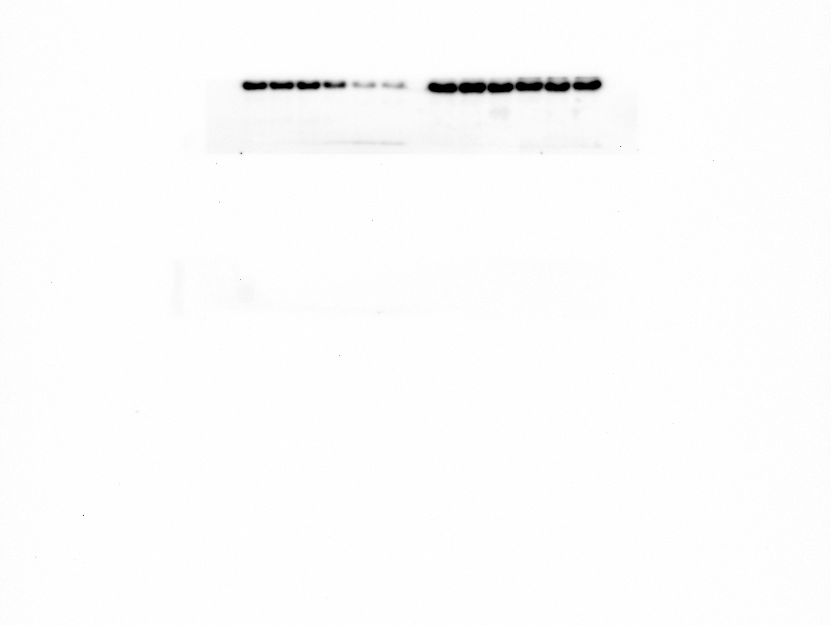

Supplement: Supplementary file 2 [file DataSheet_2.zip › Uncropped images/A2058 cells/Caspase 3.tif]

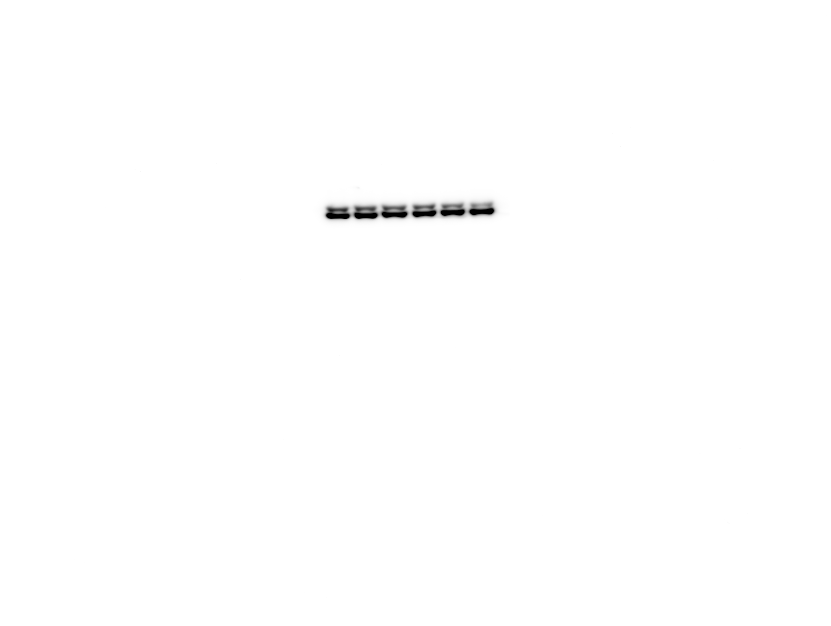

Supplement: Supplementary file 2 [file DataSheet_2.zip › Uncropped images/A2058 cells/Erk (different dosages).tif]

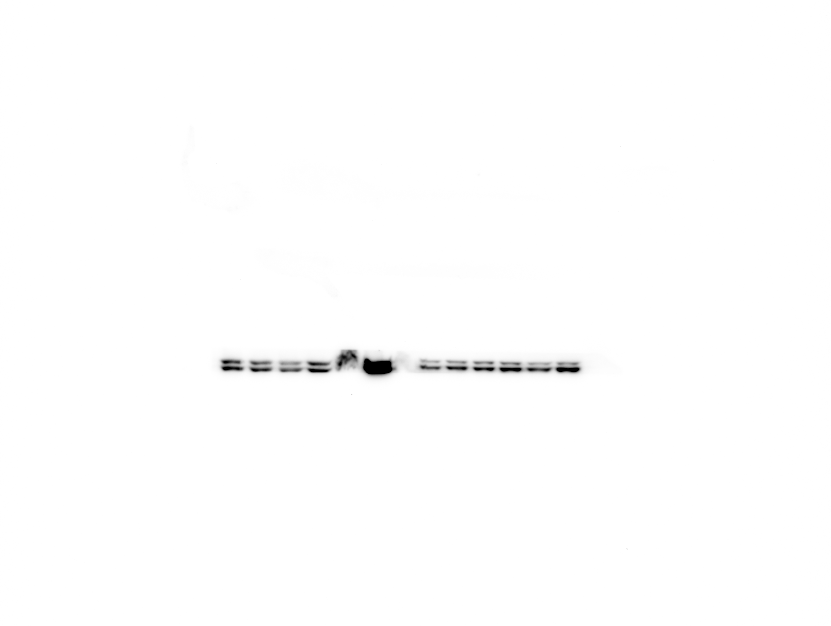

Supplement: Supplementary file 2 [file DataSheet_2.zip › Uncropped images/A2058 cells/Erk (different treatment durations).tif]

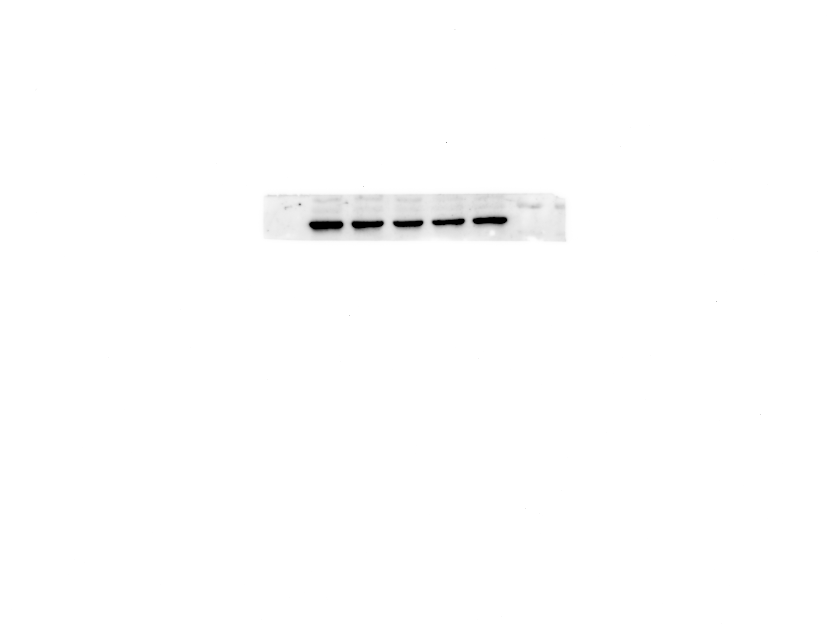

Supplement: Supplementary file 2 [file DataSheet_2.zip › Uncropped images/A2058 cells/Jak2.tif]

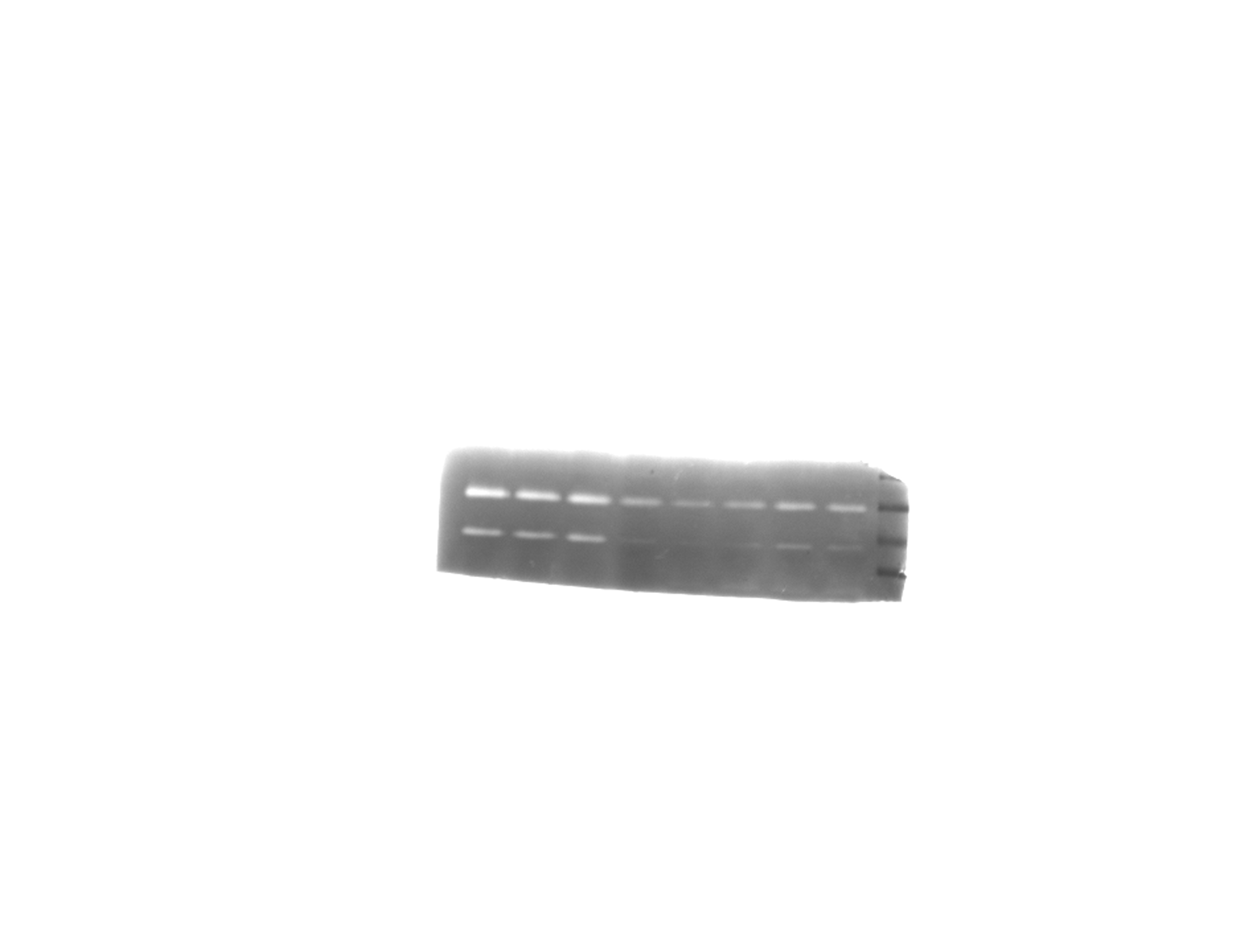

Supplement: Supplementary file 2 [file DataSheet_2.zip › Uncropped images/A2058 cells/MMP-2 and MMP-9 (Gelatin zymography).tif]

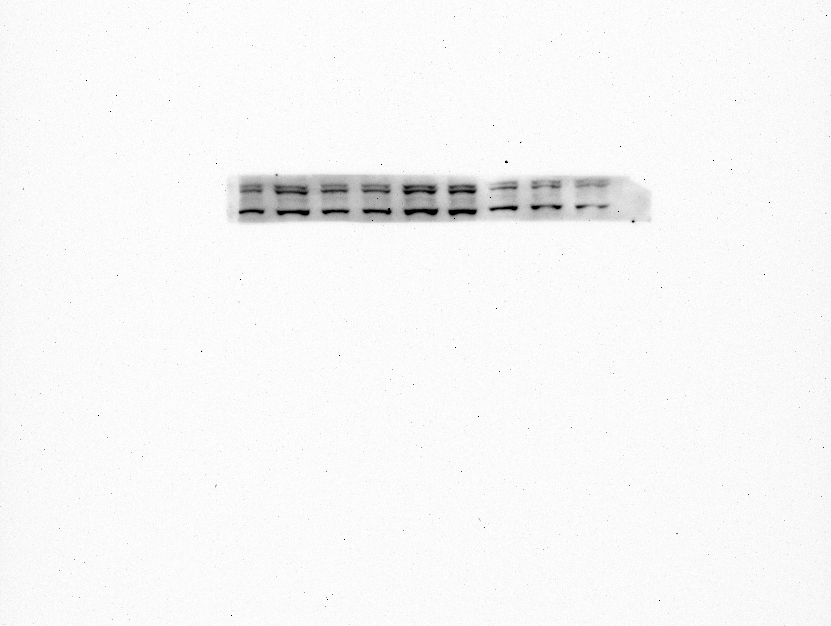

Supplement: Supplementary file 2 [file DataSheet_2.zip › Uncropped images/A2058 cells/MMP-2.tif]

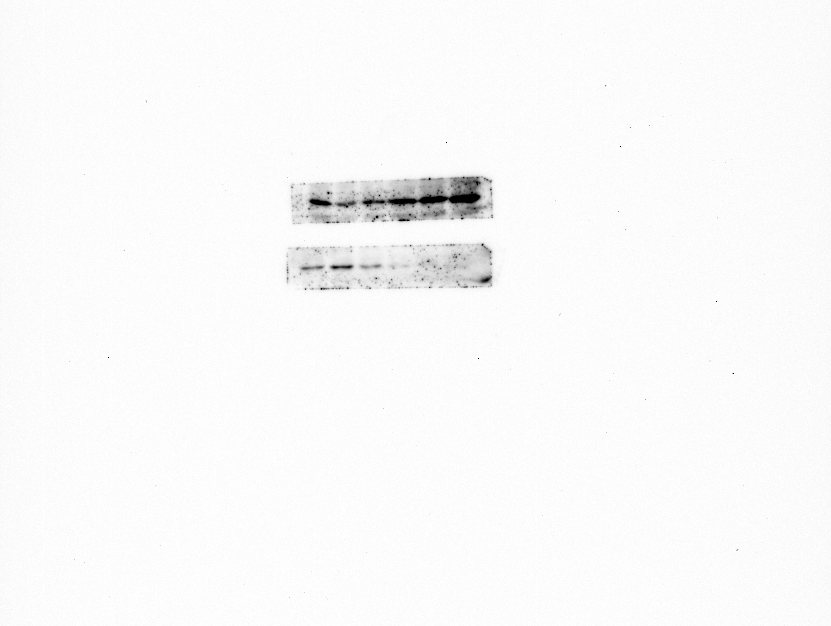

Supplement: Supplementary file 2 [file DataSheet_2.zip › Uncropped images/A2058 cells/Mcl-1.tif]

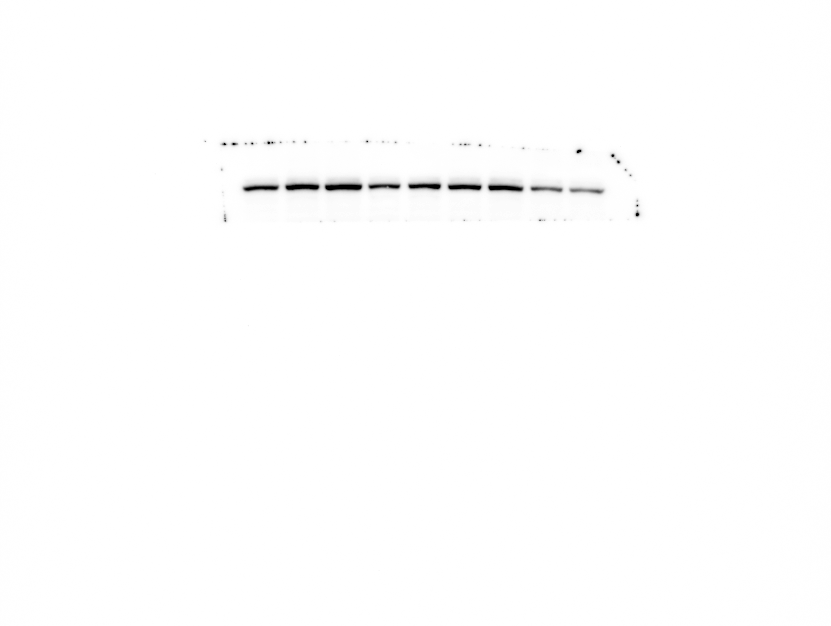

Supplement: Supplementary file 2 [file DataSheet_2.zip › Uncropped images/A2058 cells/N-cadherin.tif]

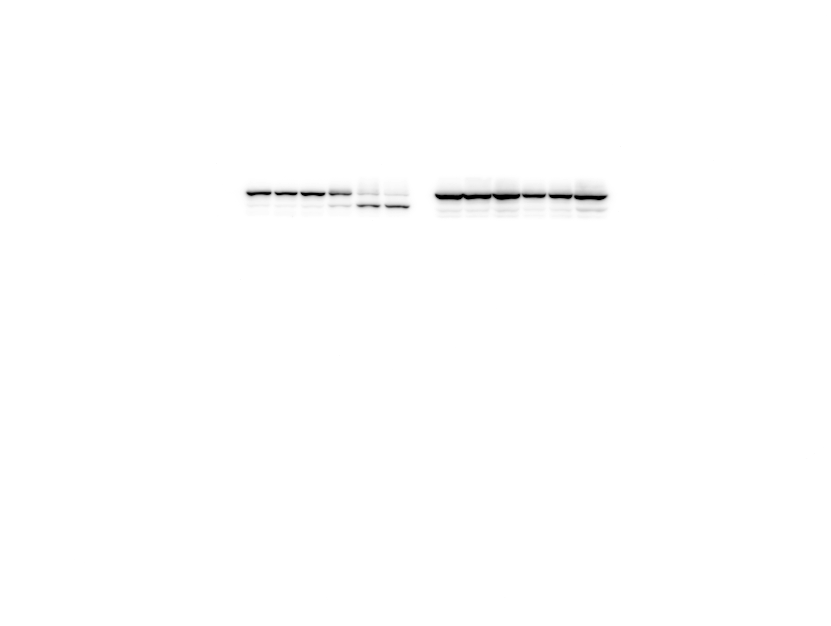

Supplement: Supplementary file 2 [file DataSheet_2.zip › Uncropped images/A2058 cells/PARP.tif]

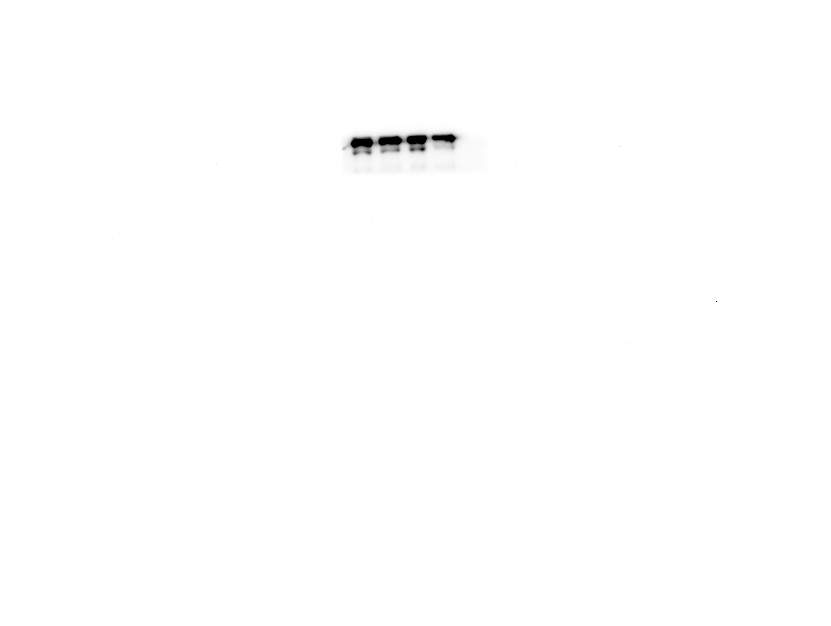

Supplement: Supplementary file 2 [file DataSheet_2.zip › Uncropped images/A2058 cells/STAT3 (cytoplasmic).tif]

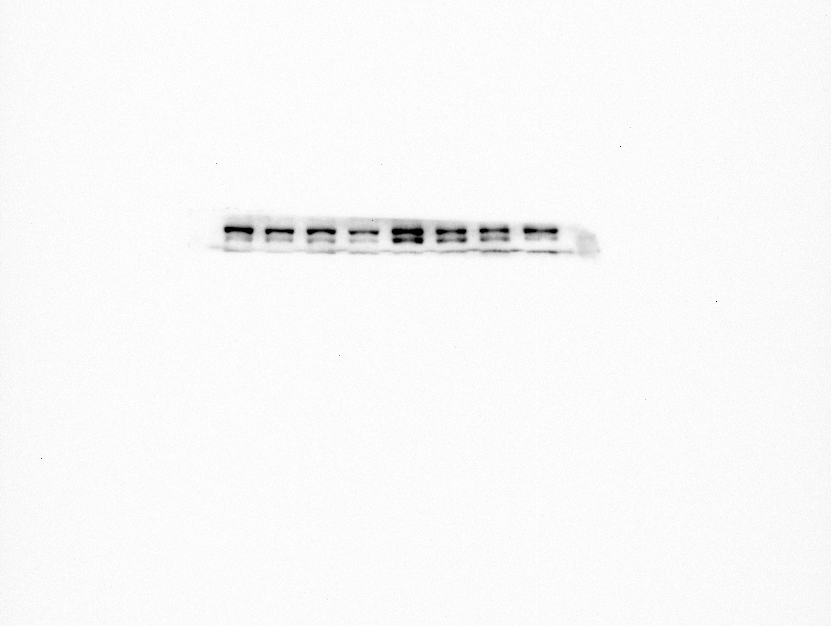

Supplement: Supplementary file 2 [file DataSheet_2.zip › Uncropped images/A2058 cells/STAT3 (nuclear).tif]

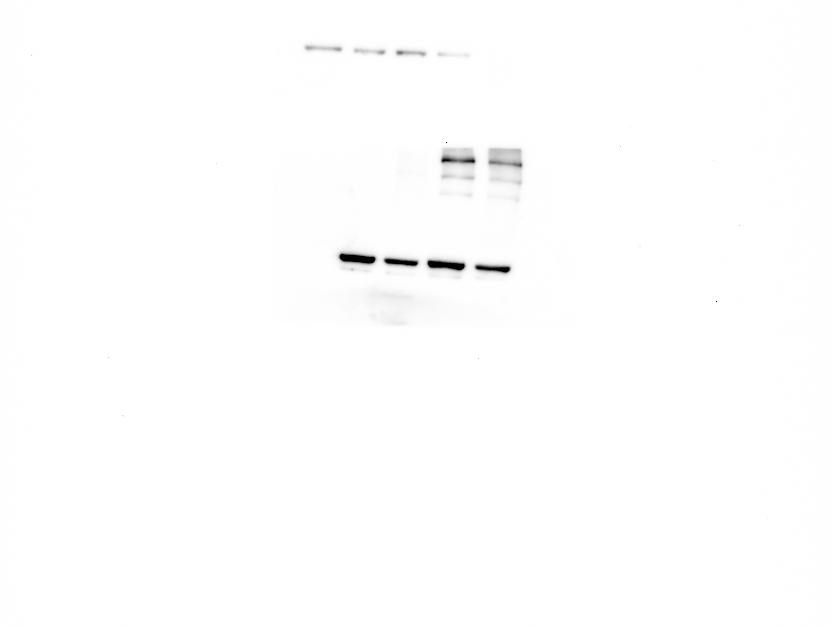

Supplement: Supplementary file 2 [file DataSheet_2.zip › Uncropped images/A2058 cells/STAT3 dimerization.tif]

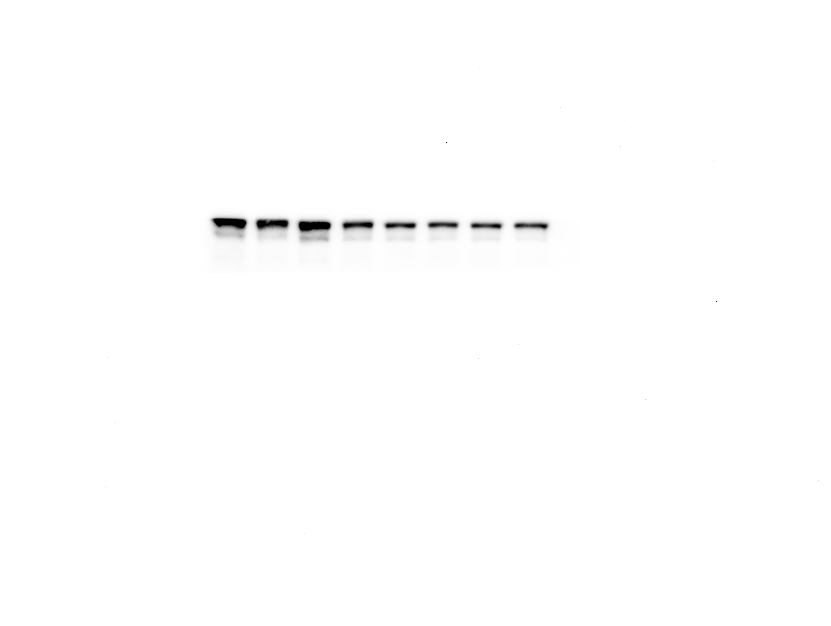

Supplement: Supplementary file 2 [file DataSheet_2.zip › Uncropped images/A2058 cells/STAT3.tif]

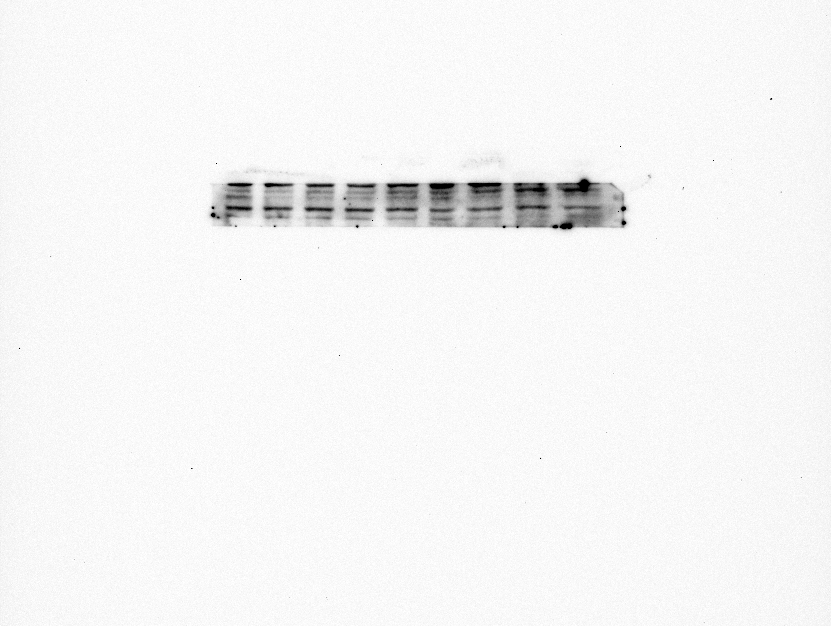

Supplement: Supplementary file 2 [file DataSheet_2.zip › Uncropped images/A2058 cells/Src.tif]

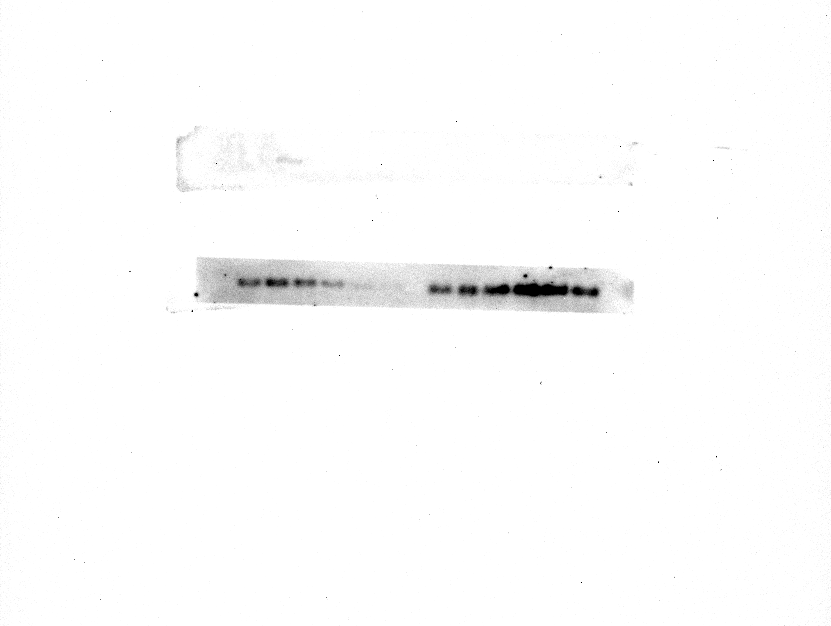

Supplement: Supplementary file 2 [file DataSheet_2.zip › Uncropped images/A2058 cells/Twist.tif]

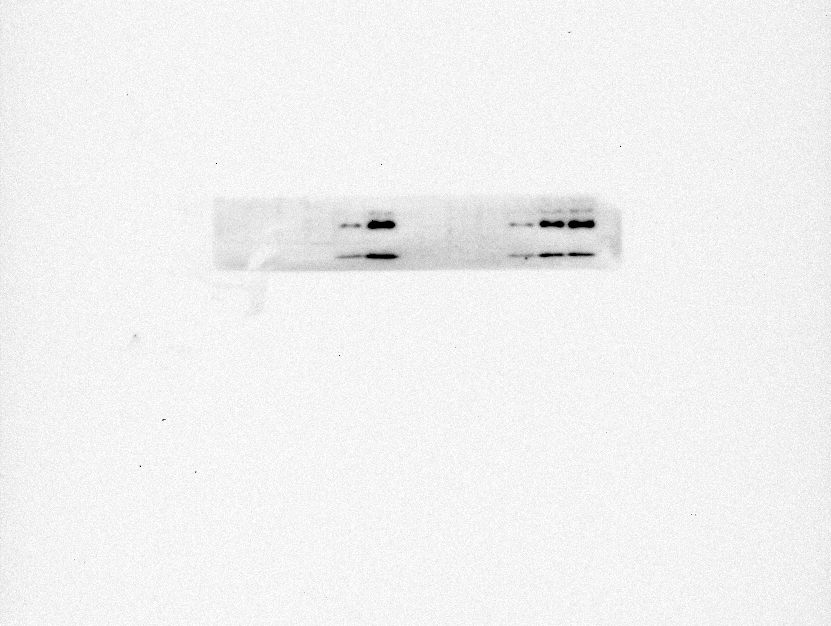

Supplement: Supplementary file 2 [file DataSheet_2.zip › Uncropped images/A2058 cells/cleaved-Caspase 3.tif]

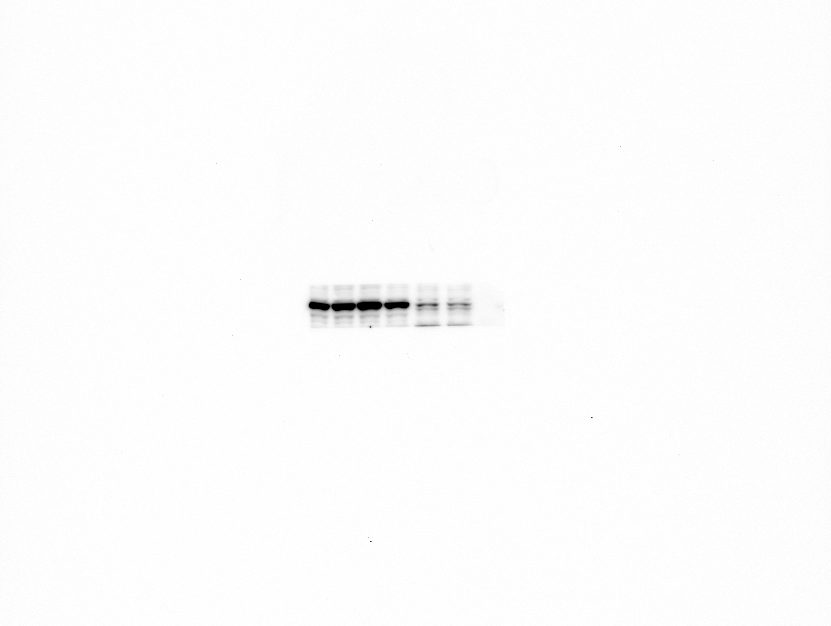

Supplement: Supplementary file 2 [file DataSheet_2.zip › Uncropped images/A2058 cells/p-Akt (different dosages).tif]

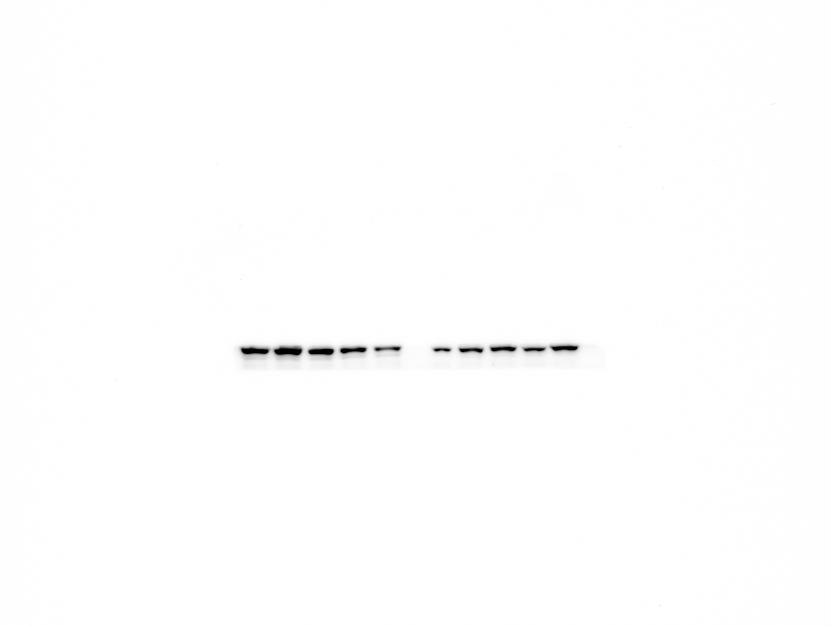

Supplement: Supplementary file 2 [file DataSheet_2.zip › Uncropped images/A2058 cells/p-Akt (different treatment durations).tif]

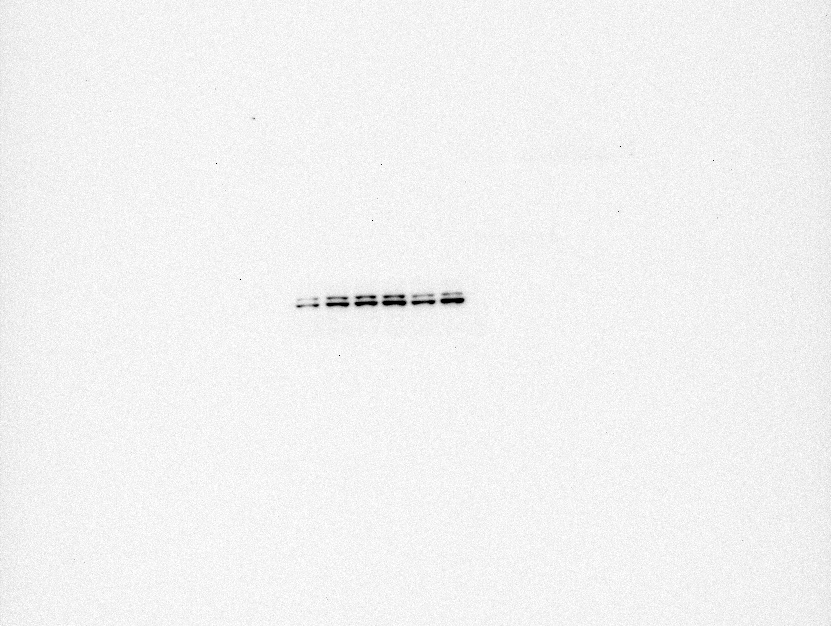

Supplement: Supplementary file 2 [file DataSheet_2.zip › Uncropped images/A2058 cells/p-Erk (different dosages).tif]

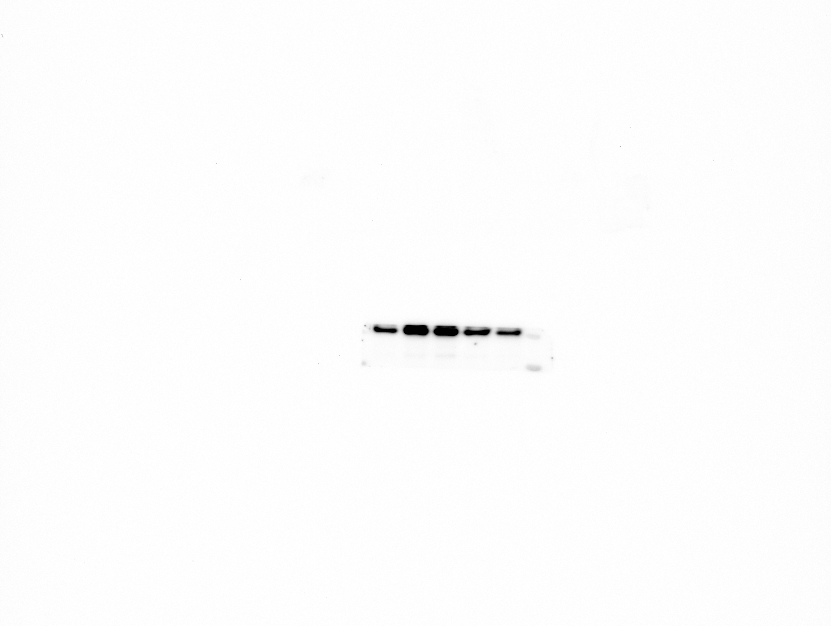

Supplement: Supplementary file 2 [file DataSheet_2.zip › Uncropped images/A2058 cells/p-Erk (different treatment durations).tif]

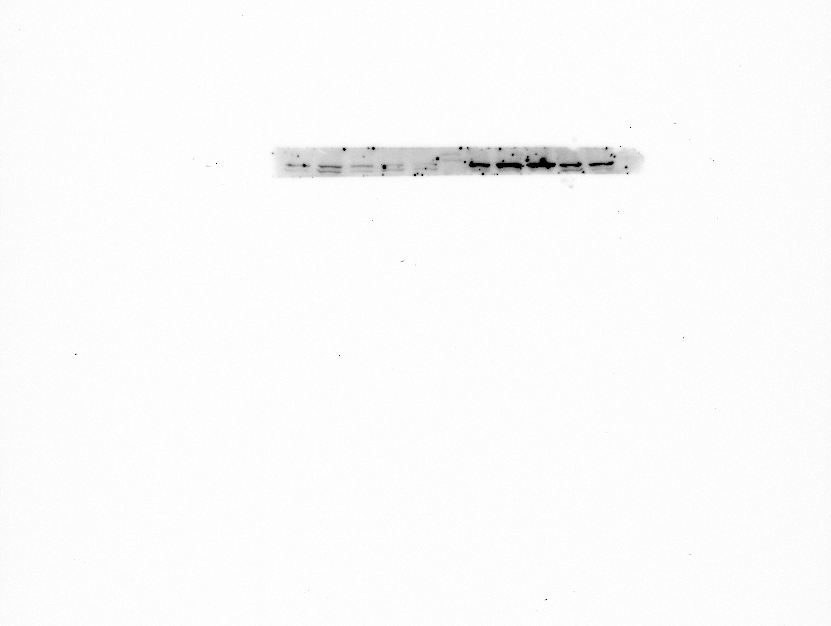

Supplement: Supplementary file 2 [file DataSheet_2.zip › Uncropped images/A2058 cells/p-Jak2.tif]

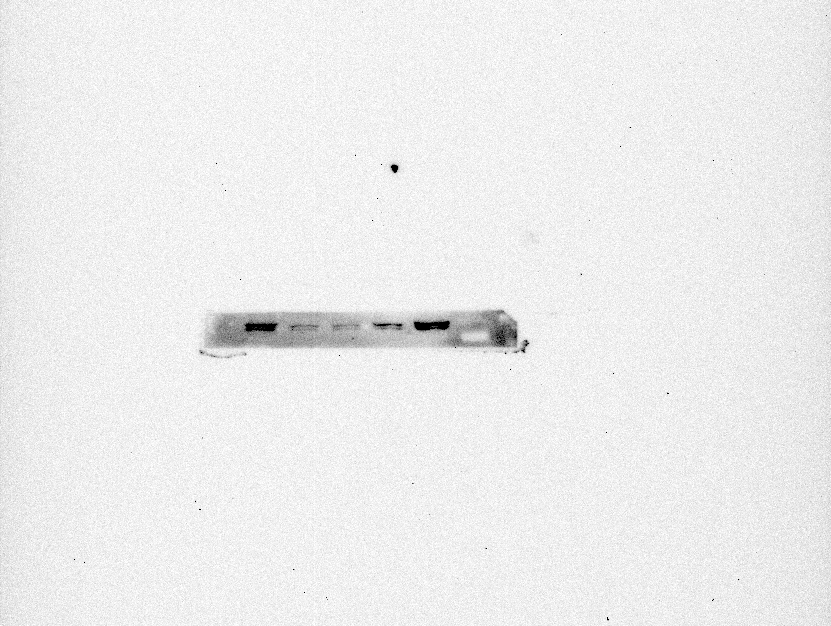

Supplement: Supplementary file 2 [file DataSheet_2.zip › Uncropped images/A2058 cells/p-STAT3.tif]

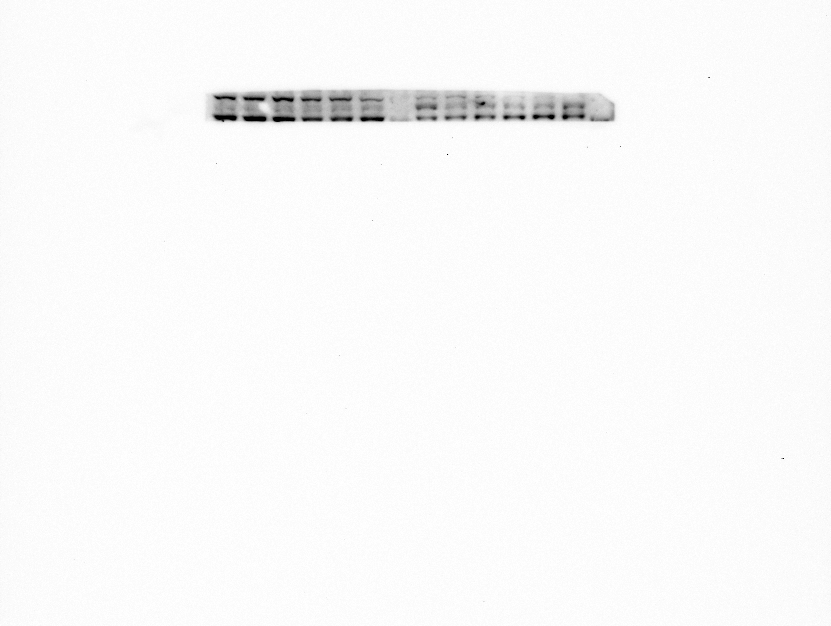

Supplement: Supplementary file 2 [file DataSheet_2.zip › Uncropped images/A2058 cells/p-Src.tif]

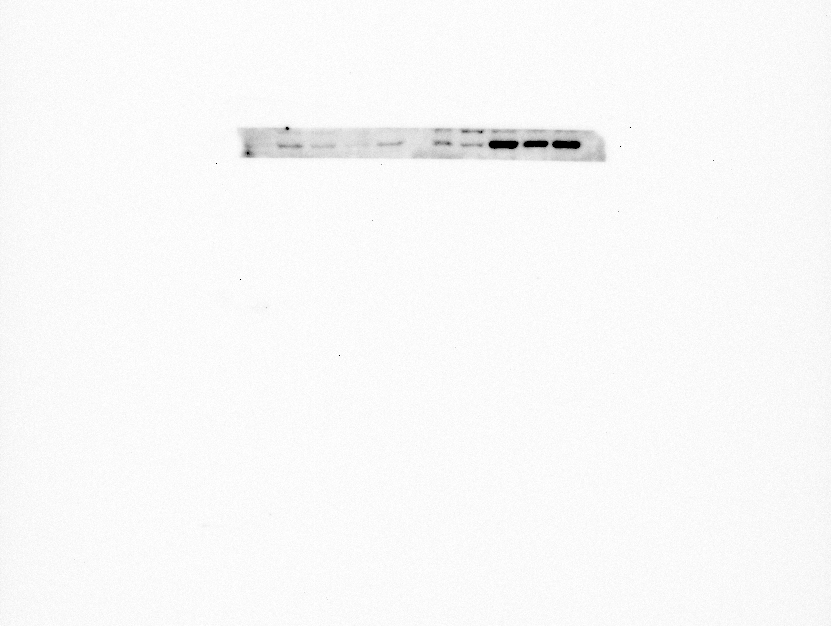

Supplement: Supplementary file 2 [file DataSheet_2.zip › Uncropped images/A2058 cells/p-p38 time 2.tif]

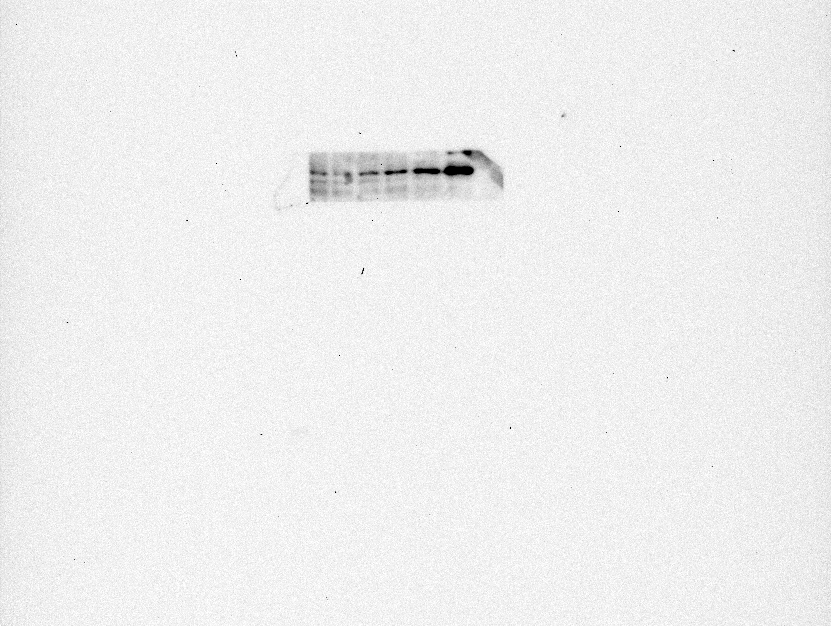

Supplement: Supplementary file 2 [file DataSheet_2.zip › Uncropped images/A2058 cells/p-p38 (different treatment durations).tif]

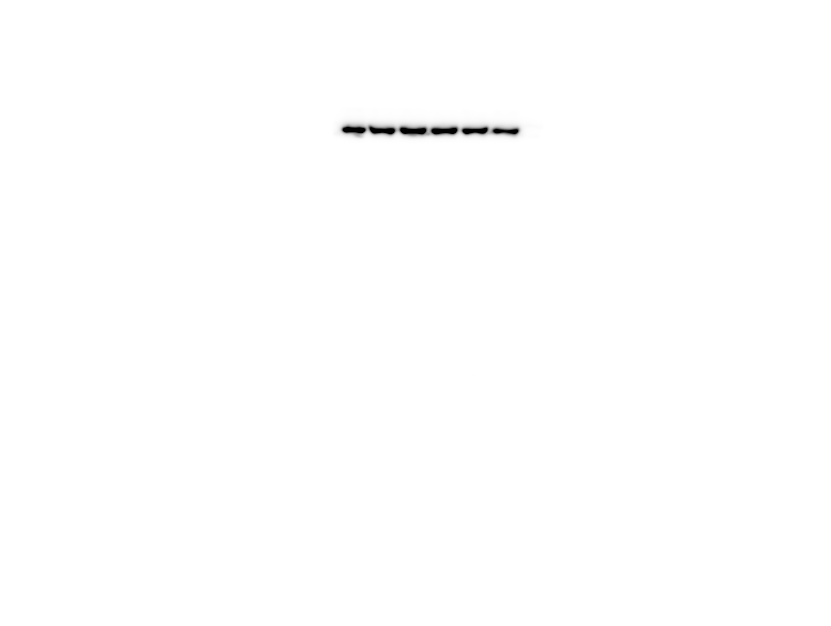

Supplement: Supplementary file 2 [file DataSheet_2.zip › Uncropped images/A2058 cells/p38 (different dosages).tif]

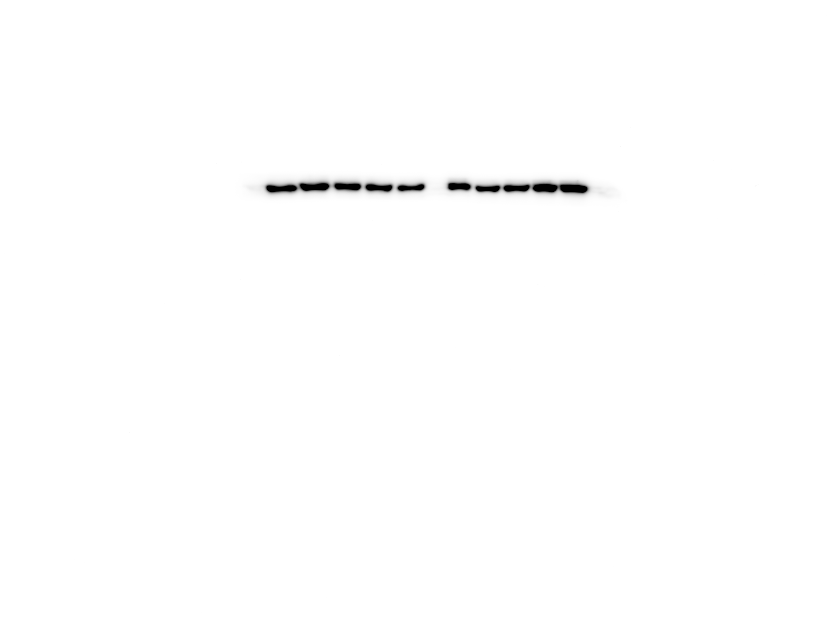

Supplement: Supplementary file 2 [file DataSheet_2.zip › Uncropped images/A2058 cells/p38 (different treatment durations).tif]

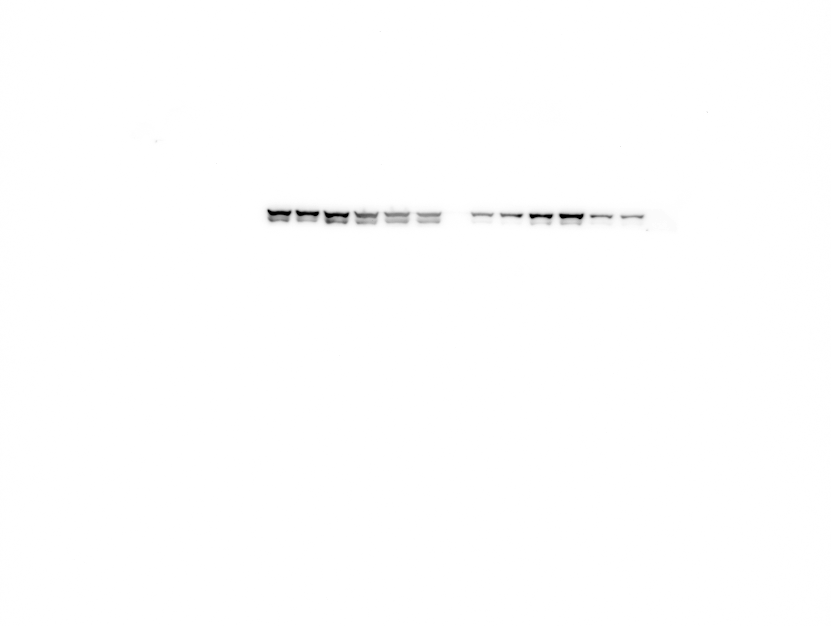

Supplement: Supplementary file 2 [file DataSheet_2.zip › Uncropped images/A2058 cells/vimentin.tif]

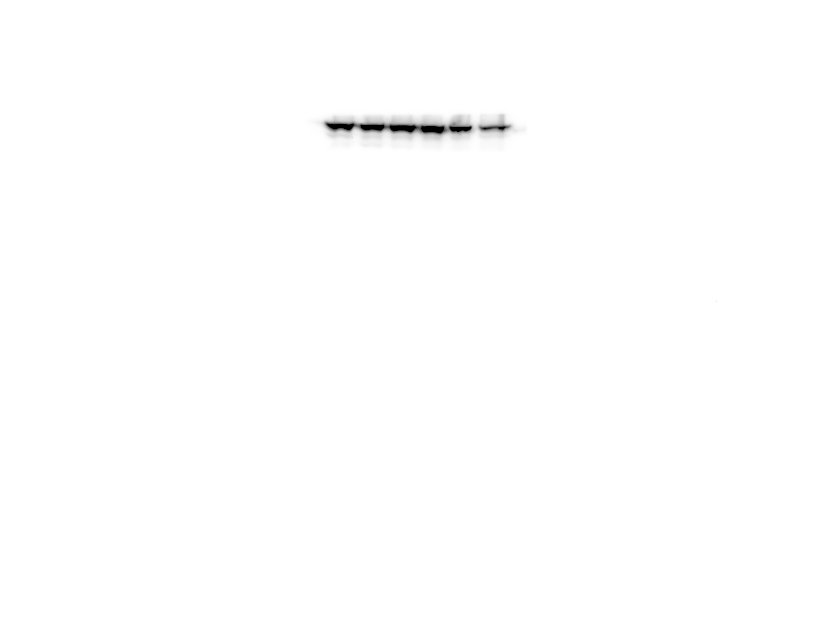

Supplement: Supplementary file 2 [file DataSheet_2.zip › Uncropped images/A375 cells/Akt (different dosages).tif]

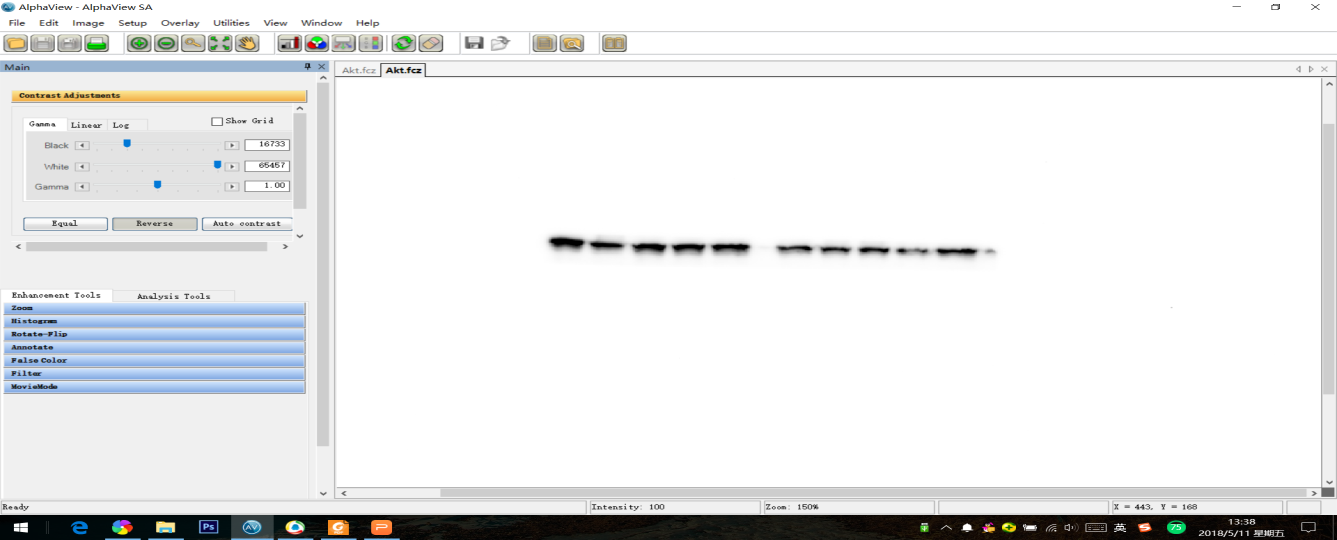

Supplement: Supplementary file 2 [file DataSheet_2.zip › Uncropped images/A375 cells/Akt (different treatment durations).tif]

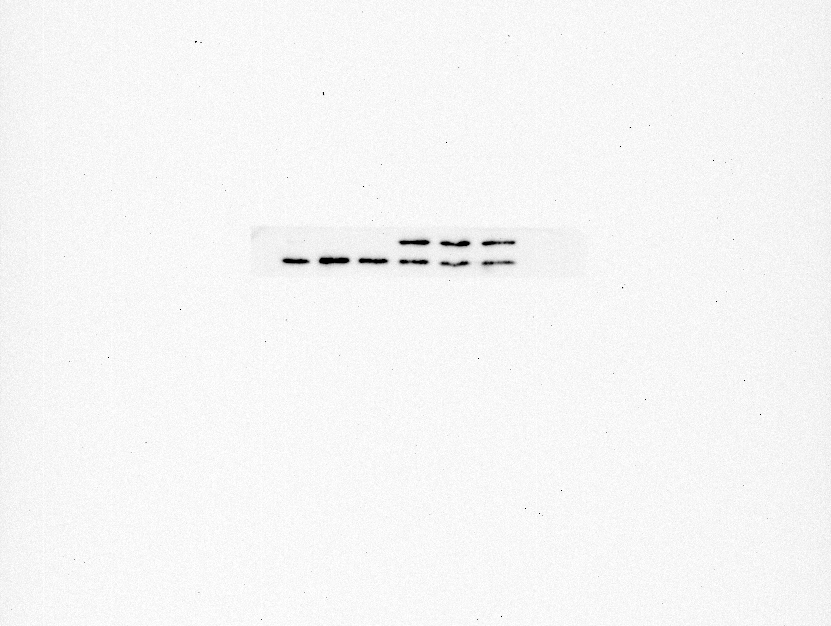

Supplement: Supplementary file 2 [file DataSheet_2.zip › Uncropped images/A375 cells/Bcl2 5.tif]

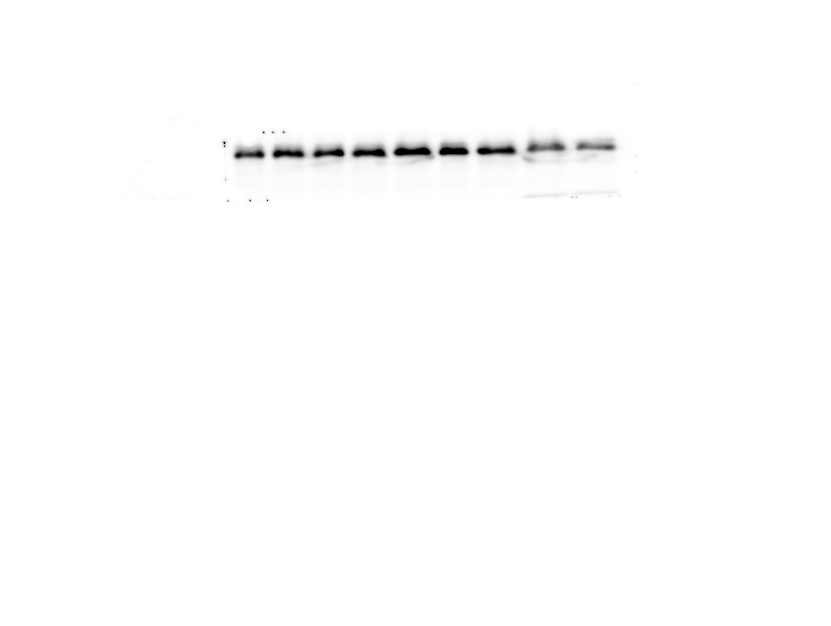

Supplement: Supplementary file 2 [file DataSheet_2.zip › Uncropped images/A375 cells/Caspase 3.tif]

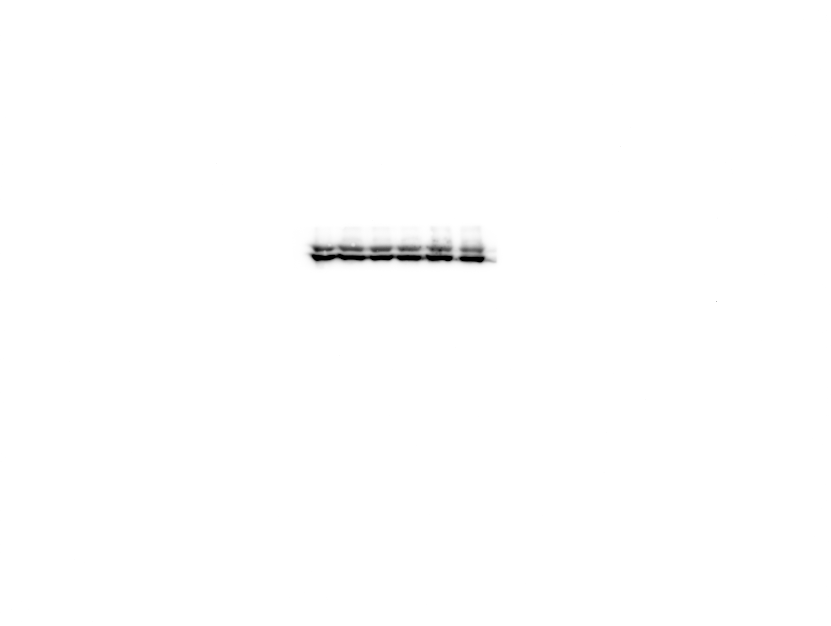

Supplement: Supplementary file 2 [file DataSheet_2.zip › Uncropped images/A375 cells/Erk (different dosages).tif]

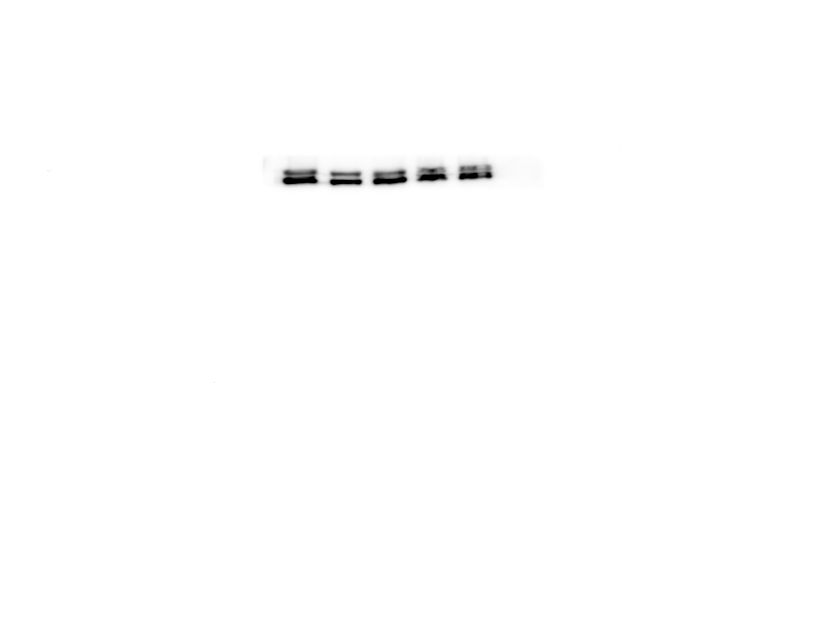

Supplement: Supplementary file 2 [file DataSheet_2.zip › Uncropped images/A375 cells/Erk (different treatment durations).tif]

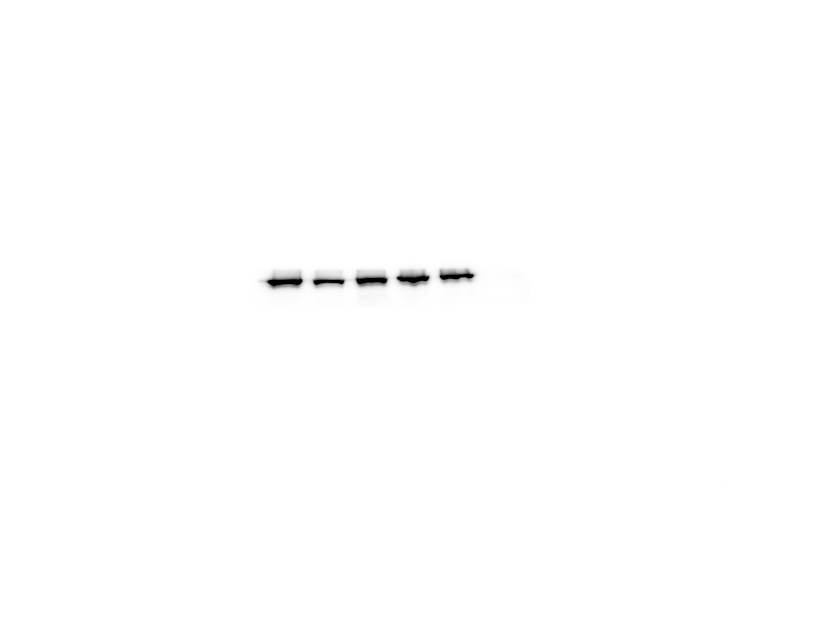

Supplement: Supplementary file 2 [file DataSheet_2.zip › Uncropped images/A375 cells/Jak2.tif]

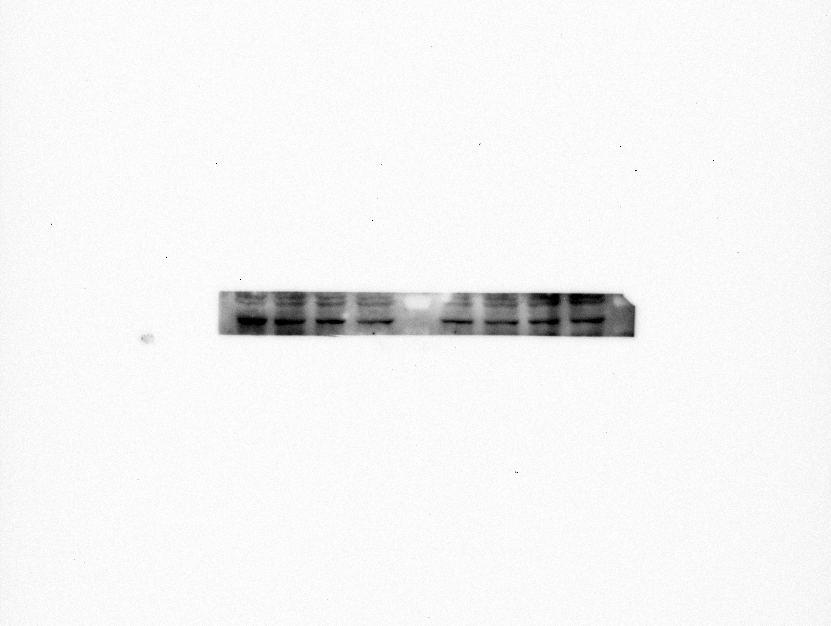

Supplement: Supplementary file 2 [file DataSheet_2.zip › Uncropped images/A375 cells/MMP-2.tif]

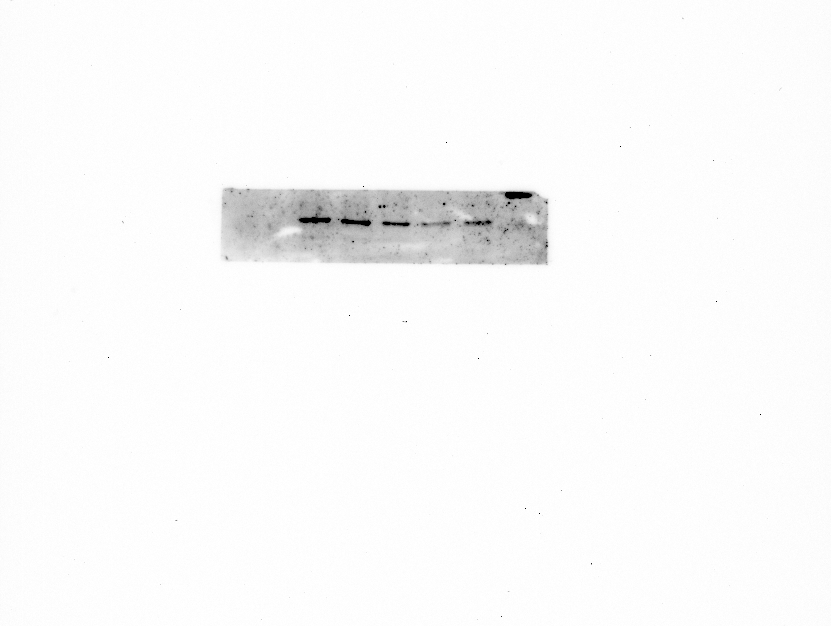

Supplement: Supplementary file 2 [file DataSheet_2.zip › Uncropped images/A375 cells/Mcl-1.tif]

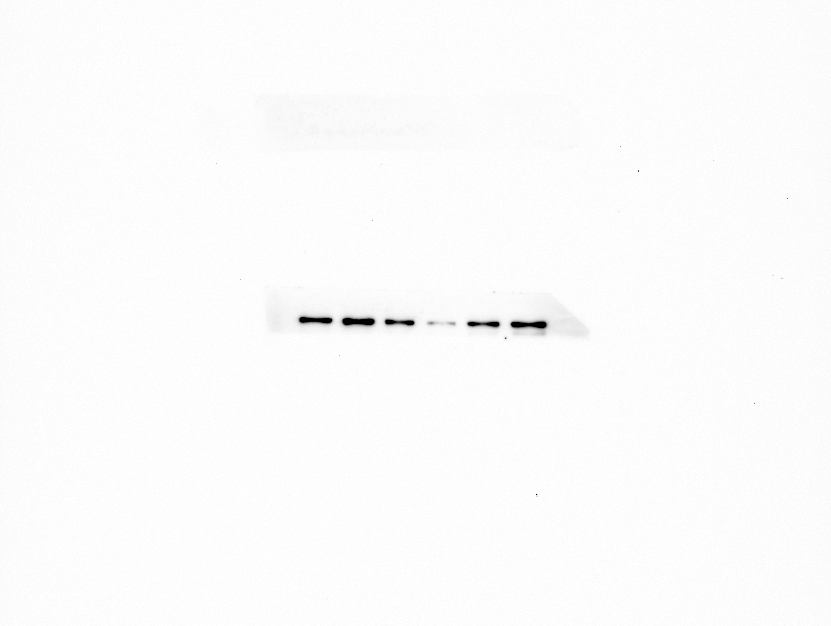

Supplement: Supplementary file 2 [file DataSheet_2.zip › Uncropped images/A375 cells/N-cadherin.tif]

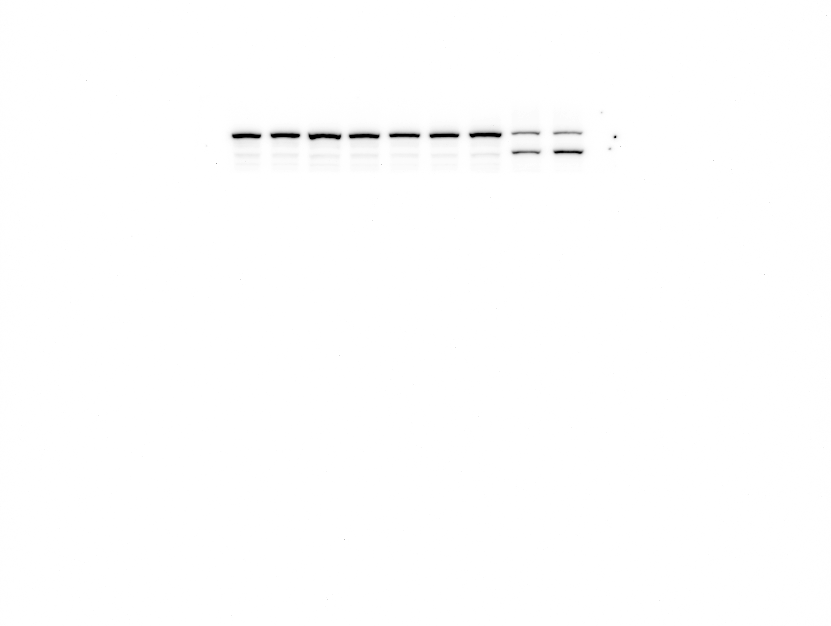

Supplement: Supplementary file 2 [file DataSheet_2.zip › Uncropped images/A375 cells/PARP.tif]

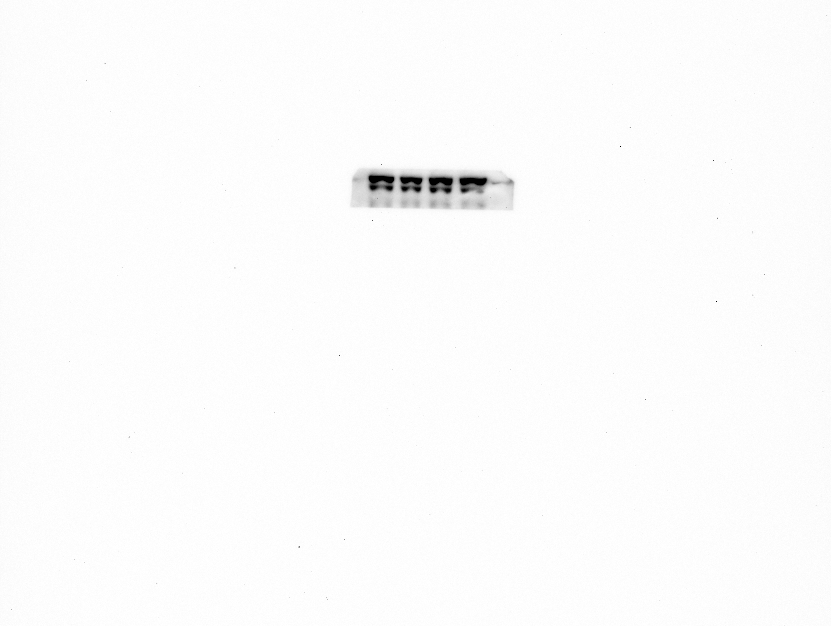

Supplement: Supplementary file 2 [file DataSheet_2.zip › Uncropped images/A375 cells/STAT3 (cytoplasmic).tif]

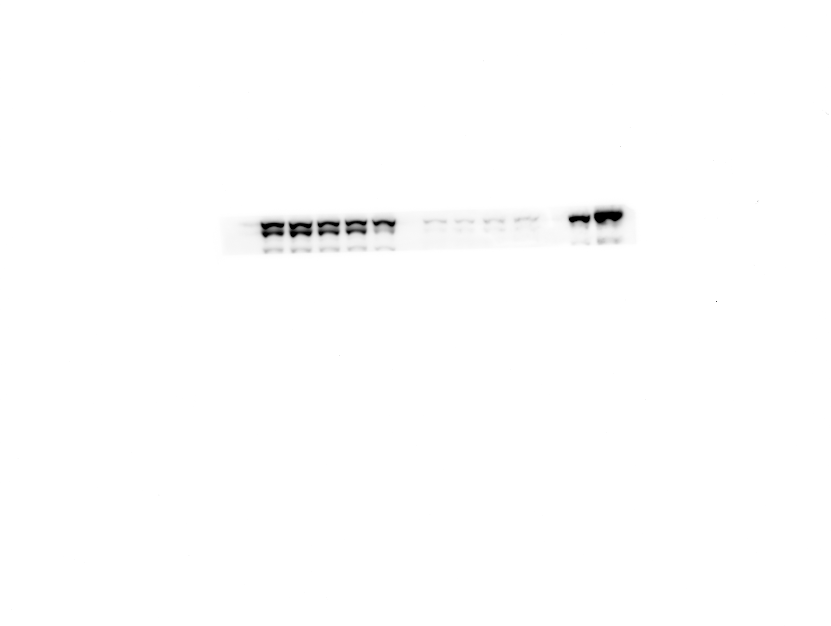

Supplement: Supplementary file 2 [file DataSheet_2.zip › Uncropped images/A375 cells/STAT3 (overexpression of STAT3 in A375 cells).tif]

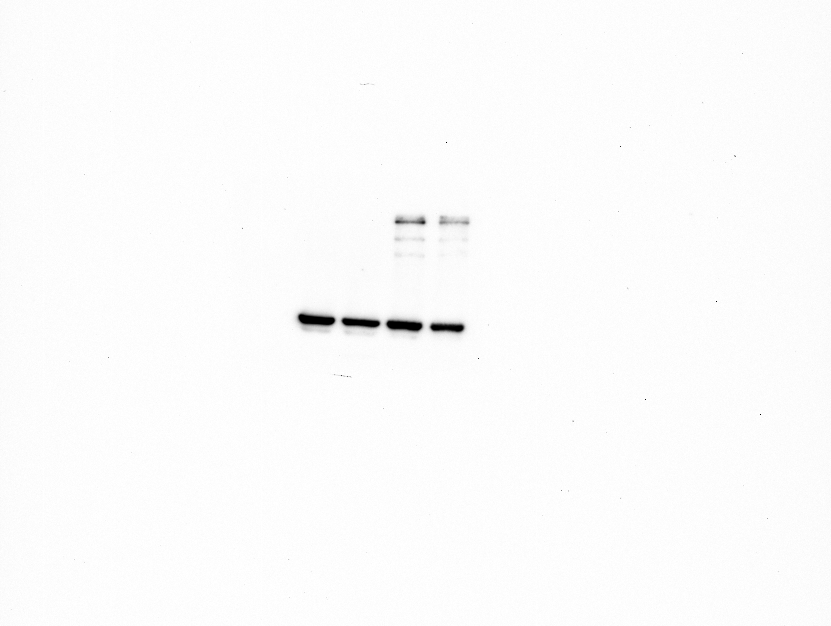

Supplement: Supplementary file 2 [file DataSheet_2.zip › Uncropped images/A375 cells/STAT3 dimerization.tif]

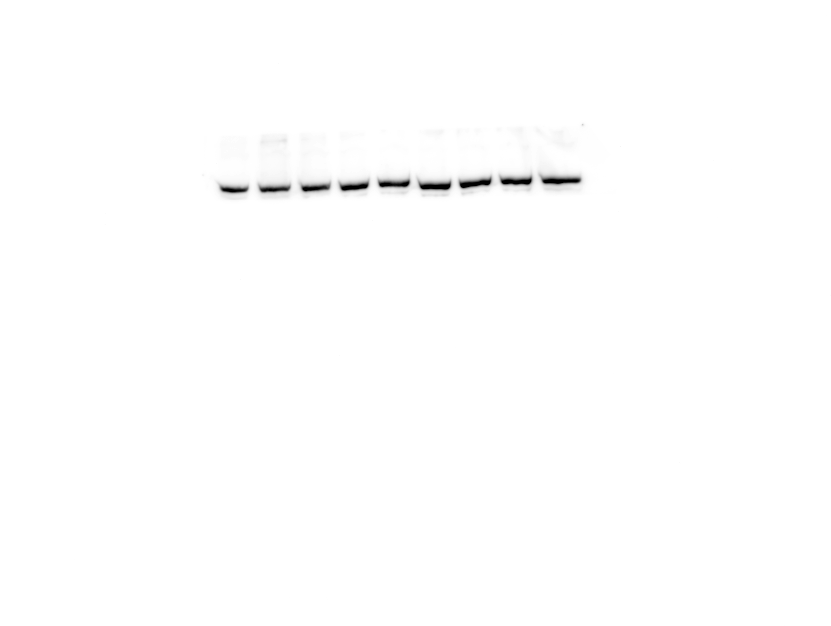

Supplement: Supplementary file 2 [file DataSheet_2.zip › Uncropped images/A375 cells/STAT3.tif]

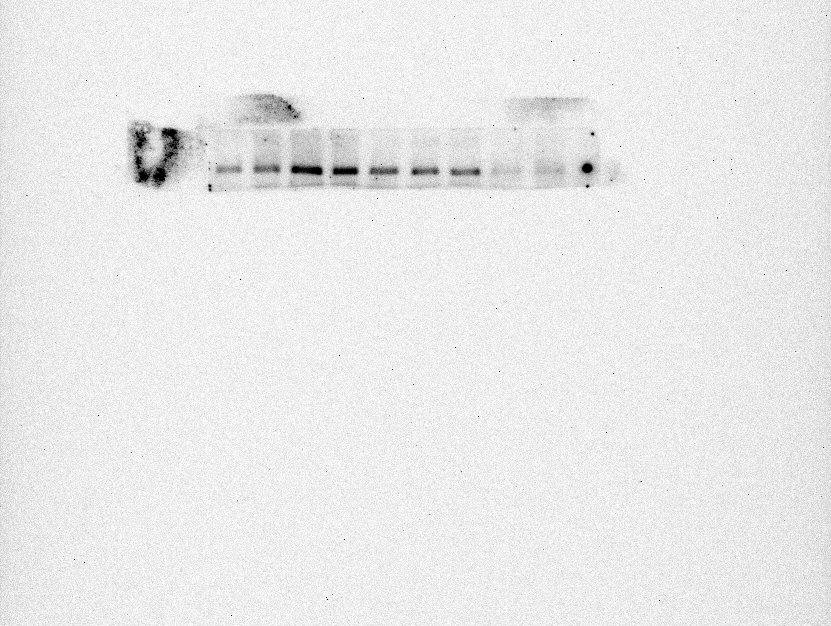

Supplement: Supplementary file 2 [file DataSheet_2.zip › Uncropped images/A375 cells/Twist.tif]

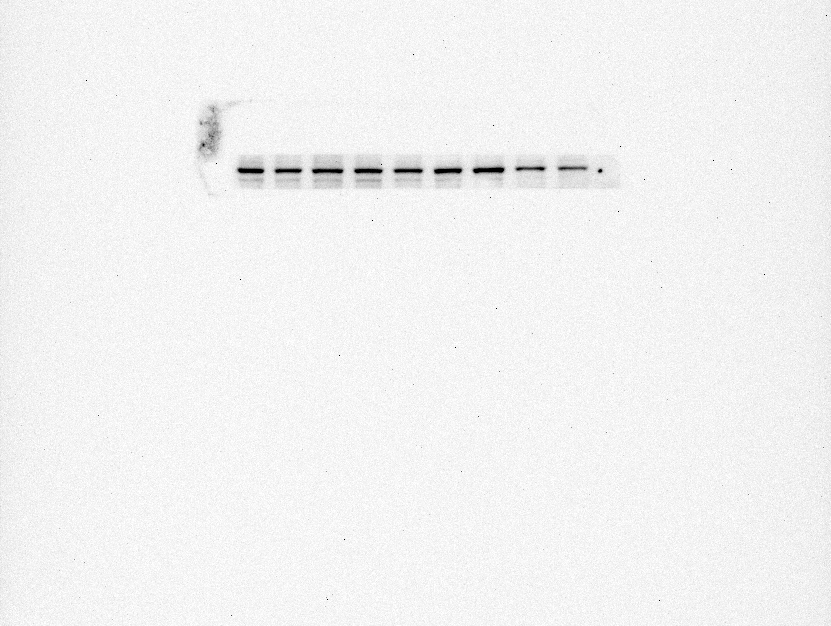

Supplement: Supplementary file 2 [file DataSheet_2.zip › Uncropped images/A375 cells/p-Akt (different dosages).tif]

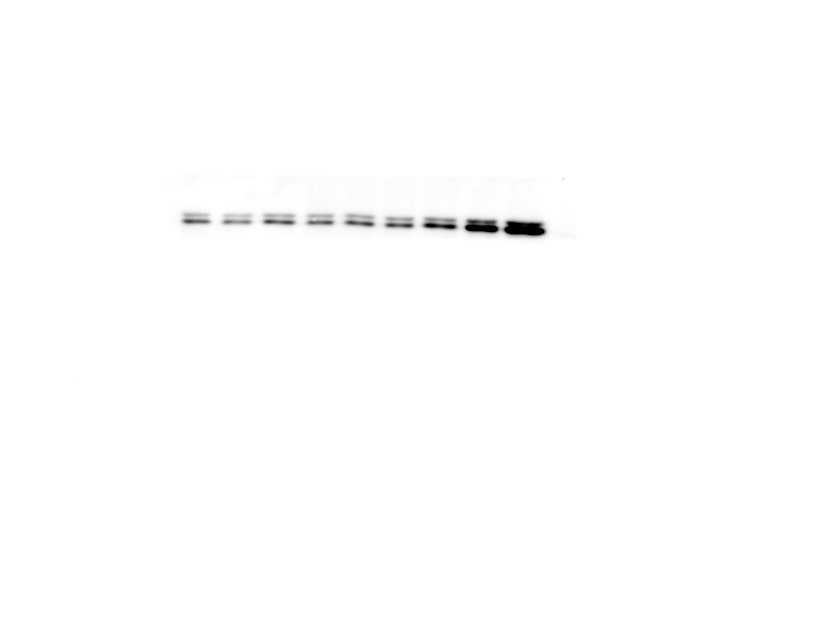

Supplement: Supplementary file 2 [file DataSheet_2.zip › Uncropped images/A375 cells/p-Erk (different dosages).tif]

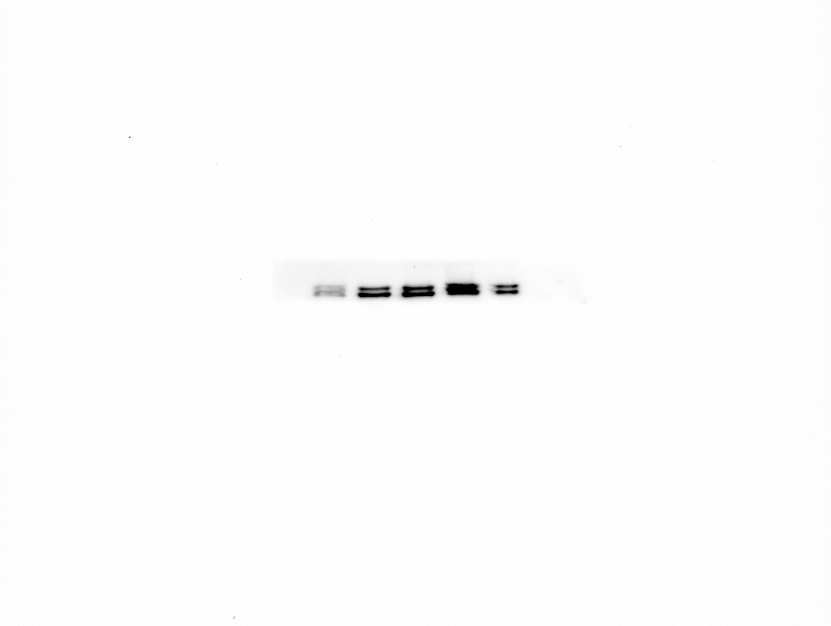

Supplement: Supplementary file 2 [file DataSheet_2.zip › Uncropped images/A375 cells/p-Erk (different treatment durations).tif]

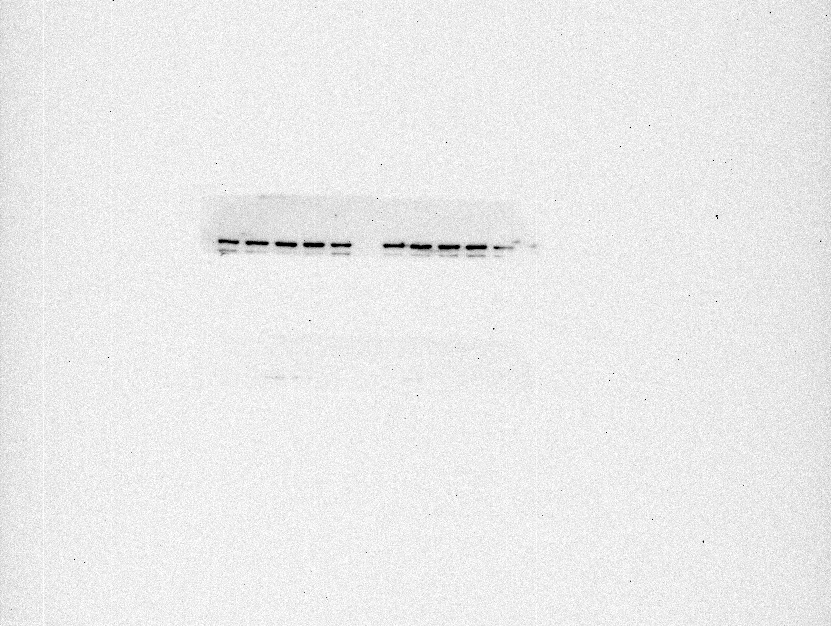

Supplement: Supplementary file 2 [file DataSheet_2.zip › Uncropped images/A375 cells/p-Jak2.tif]

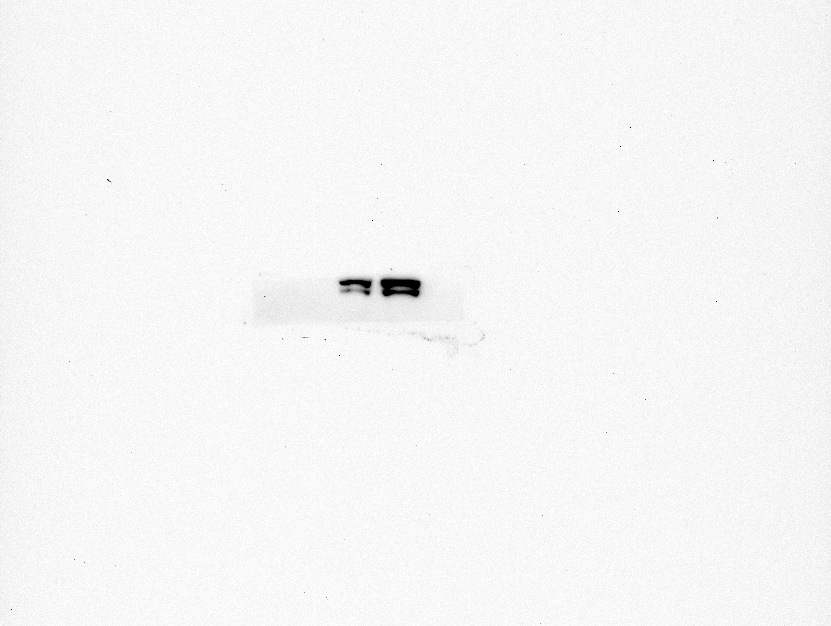

Supplement: Supplementary file 2 [file DataSheet_2.zip › Uncropped images/A375 cells/p-STAT3 (overexpression of STAT3 in A375 cells).tif]

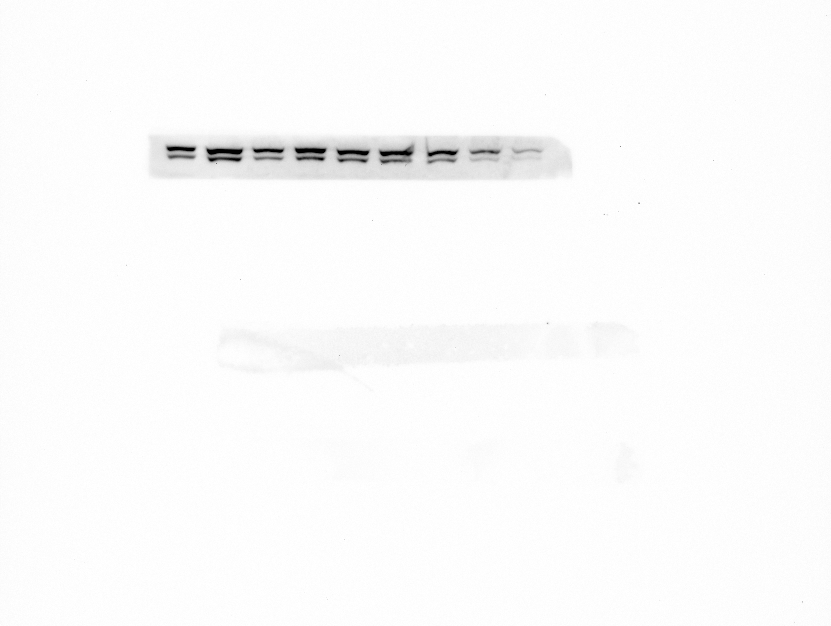

Supplement: Supplementary file 2 [file DataSheet_2.zip › Uncropped images/A375 cells/p-STAT3.tif]

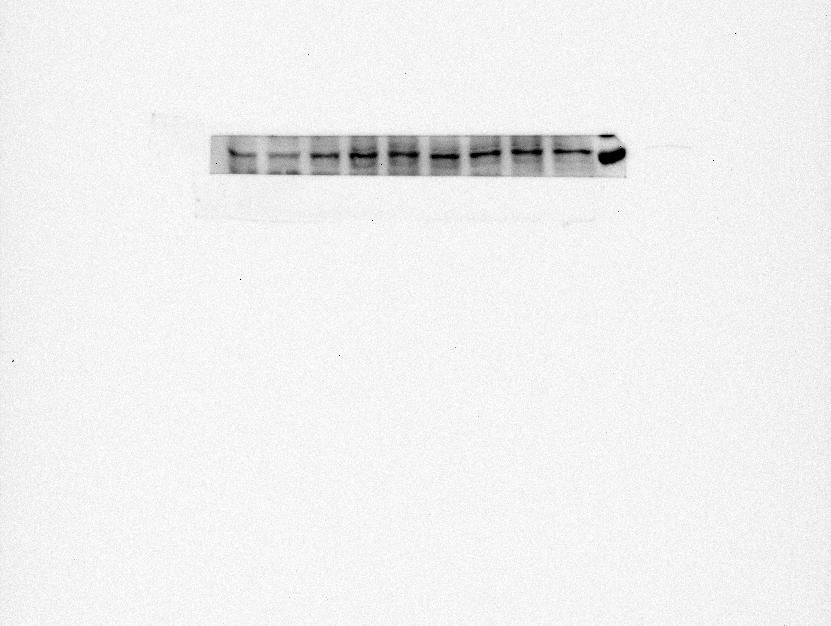

Supplement: Supplementary file 2 [file DataSheet_2.zip › Uncropped images/A375 cells/p-Src.tif]

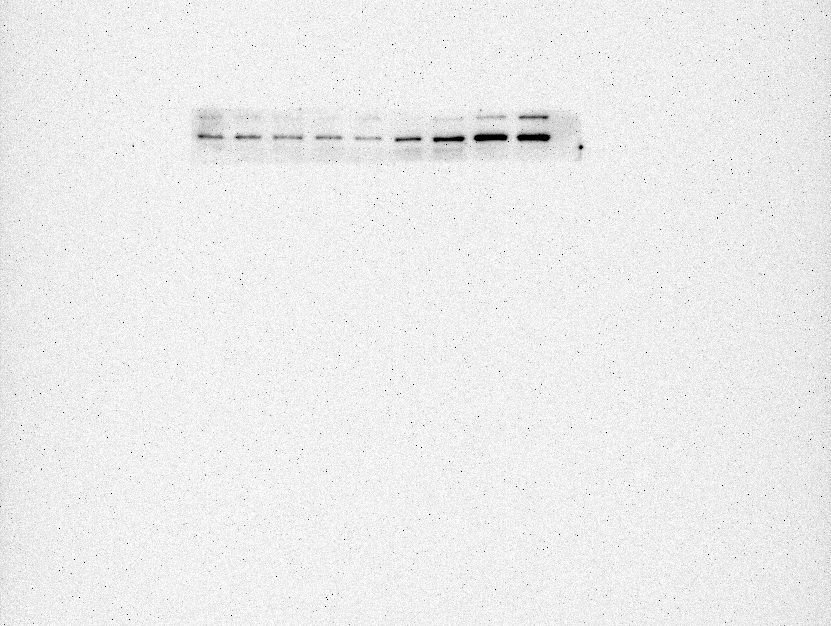

Supplement: Supplementary file 2 [file DataSheet_2.zip › Uncropped images/A375 cells/p-p38 (different dosages).tif]

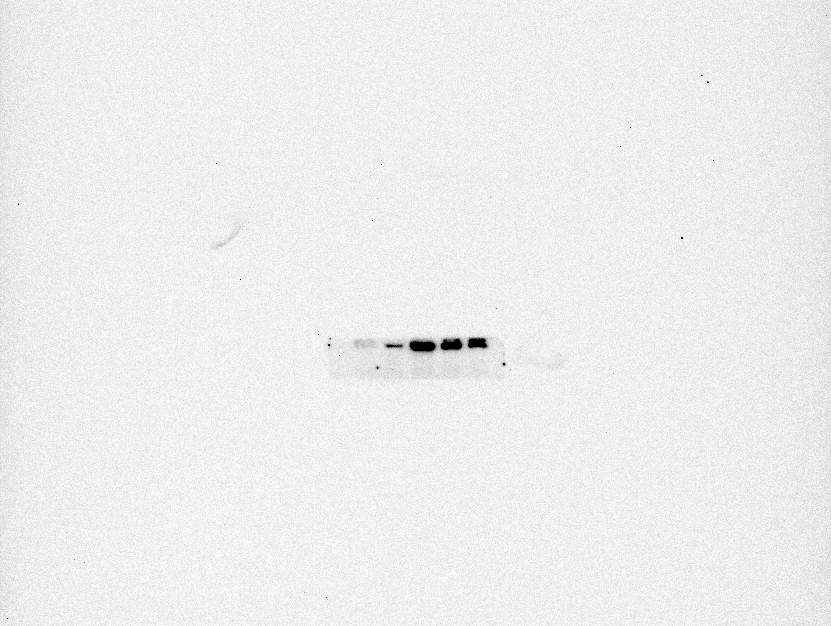

Supplement: Supplementary file 2 [file DataSheet_2.zip › Uncropped images/A375 cells/p-p38 (different treatment durations).tif]

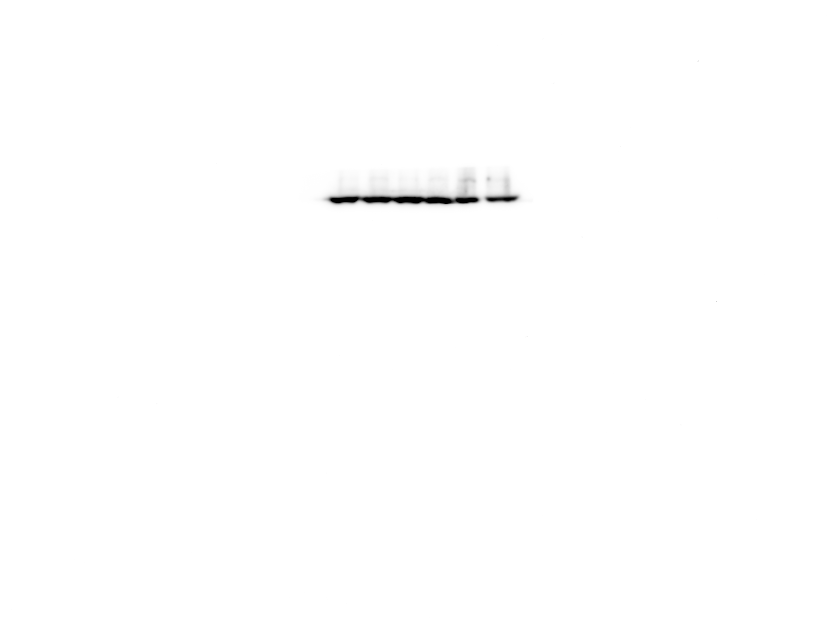

Supplement: Supplementary file 2 [file DataSheet_2.zip › Uncropped images/A375 cells/p38 (different dosages).tif]

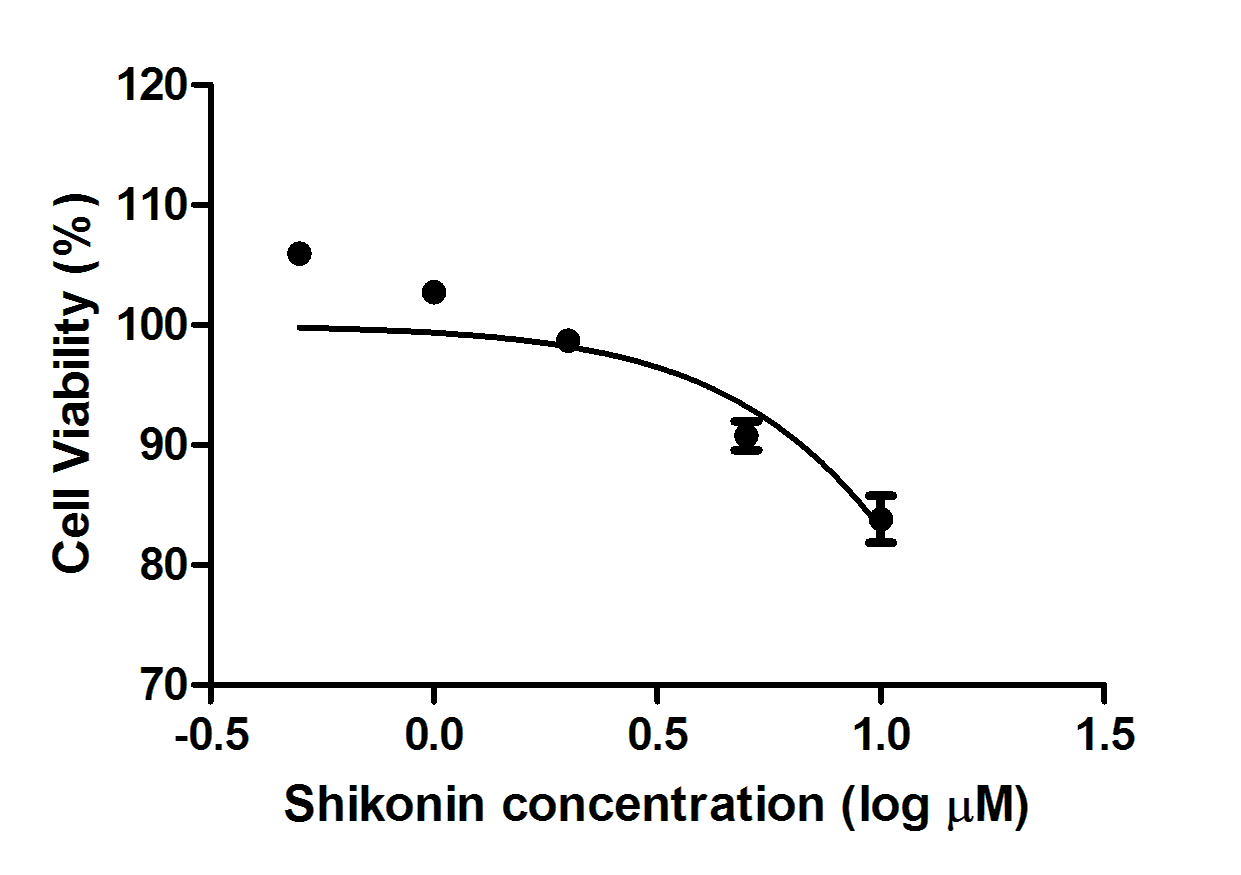

Supplement: Supplementary file 3 [file Image_1.tif]

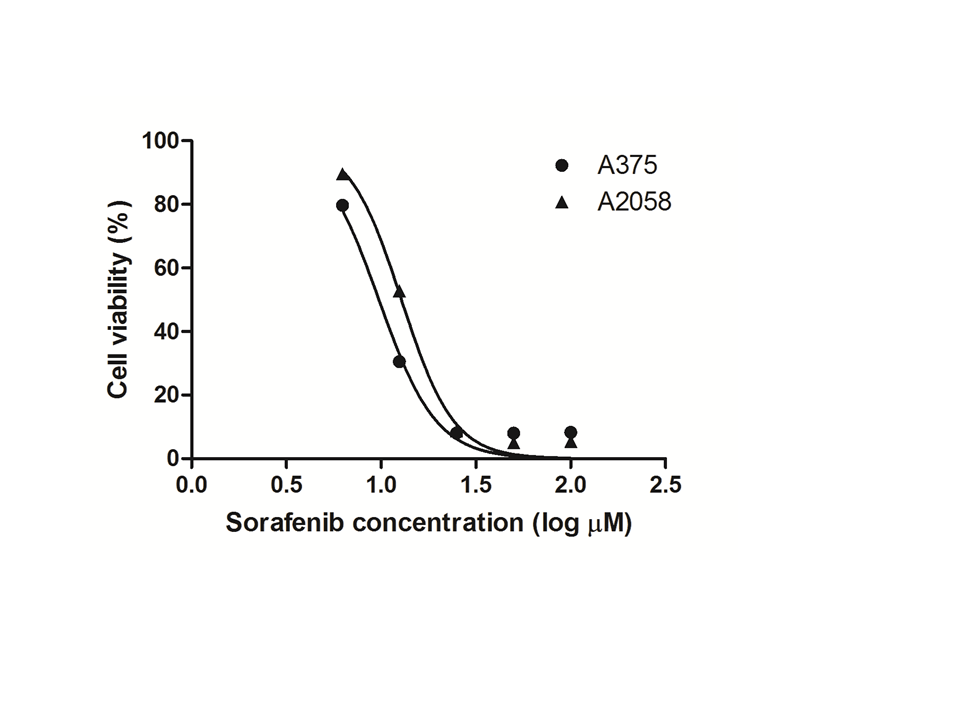

Supplement: Supplementary file 4 [file Image_2.tif]

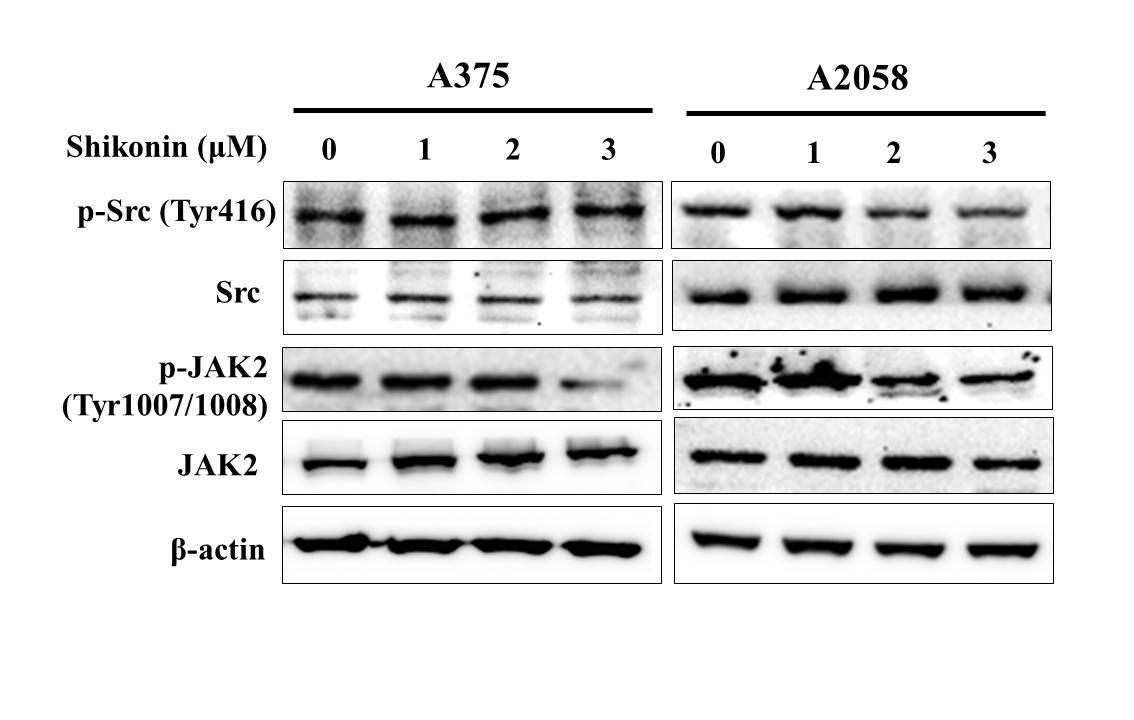

Supplement: Supplementary file 5 [file Image_3.tif]

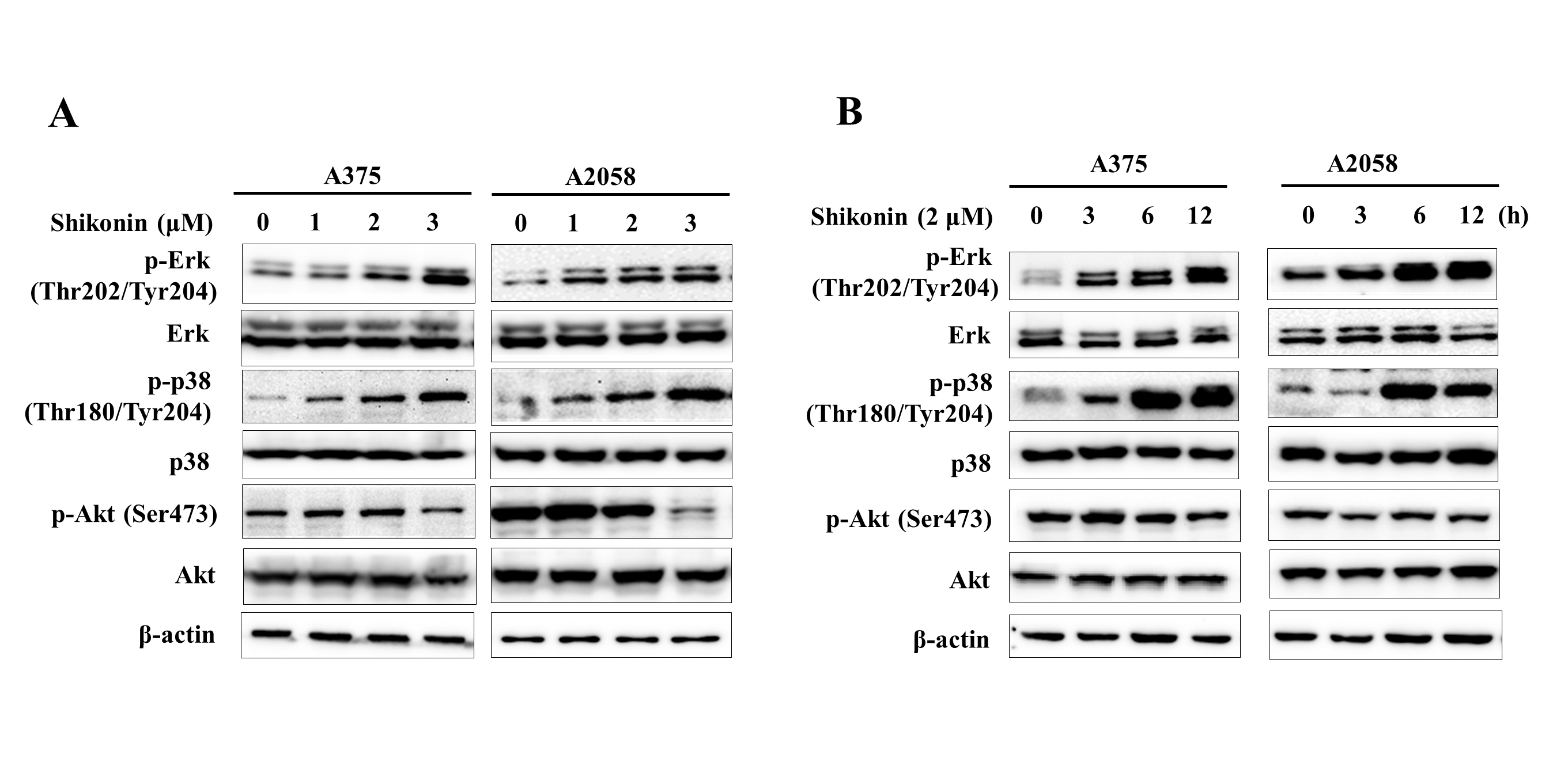

Supplement: Supplementary file 6 [file Image_4.tif]
